# Supplementary material for: Cross-Packaging and Capsid Mosaic Formation in Multiplexed AAV Libraries
Source: Mol Ther Methods Clin Dev. 2019 Nov 26;17:107–21. doi: 10.1016/j.omtm.2019.11.014 (PMC6938944; doi:10.1016/j.omtm.2019.11.014)
Supplement: Document S1. Figures S1–S11 and Tables S1–S5 [file mmc1.pdf]

OMTM, Volume *17*

Supplemental Information

Cross-Packaging and Capsid Mosaic Formation in Multiplexed AAV Libraries

Pauline F. Schmit, Simon Pacouret, Eric Zinn, Elizabeth Telford, Fotini Nicolaou, Frédéric Broucque, Eva Andres-Mateos, Ru Xiao, Magalie Penaud-Budloo, Mohammed Bouzelha, Nicolas Jaulin, Oumeya Adjali, Eduard Ayuso, and Luk H. Vandenberghe

Supplemental Material

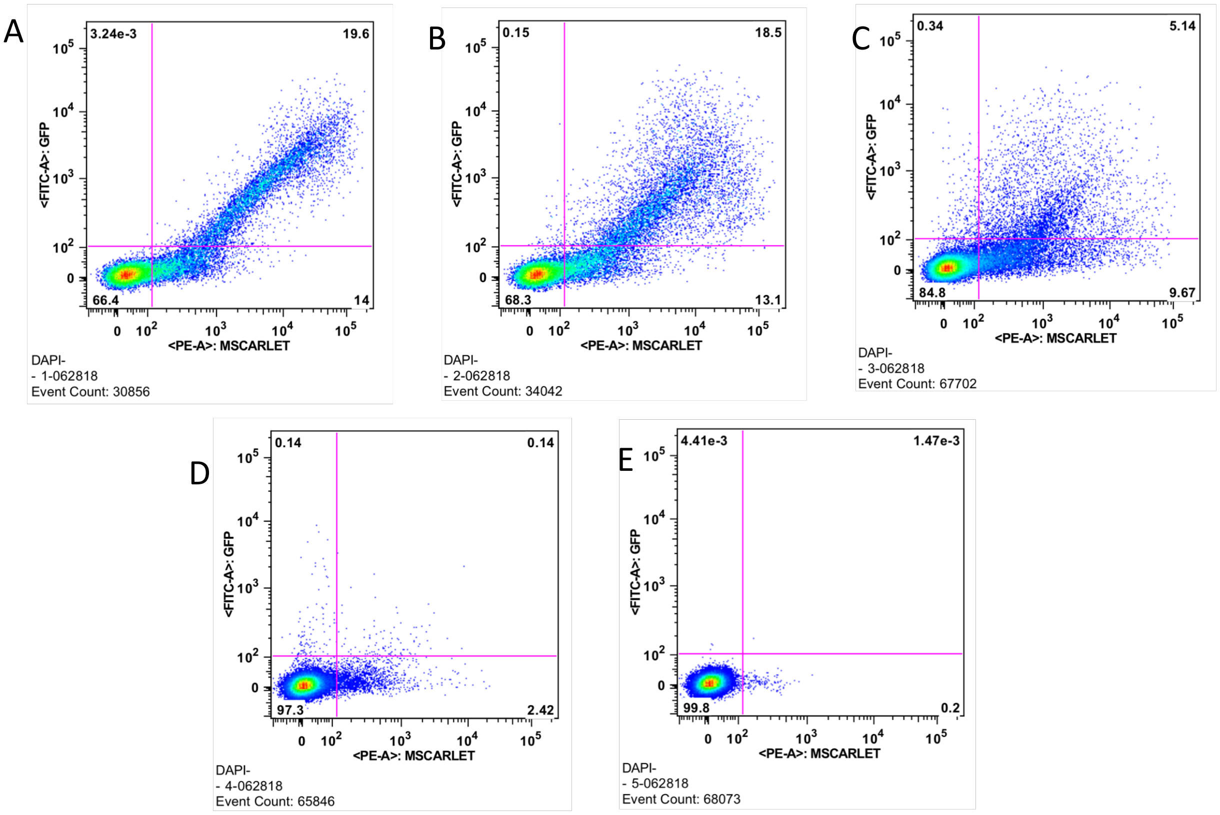

Figure S1: Flow cytometry of dually-transfected cell populations. A. HEK293 cells co-transfected with 1.3 ug each of ITR-flanked CMV.eGFP plasmid, CMV. mScarlet plasmid, and ITR-free AAV2/8 Rep/Cap production plasmids, in addition to 2.6 ug deltaF6 and a promoter-free dummy plasmid to normalize total DNA transfected. Cells were harvested after 48 hours and subjected to flow cytometry. Plots shown consist of single live cells. **B.** Cells transfected and analyzed as in **A**, but with 390 ng total AAV8/eGFP/mScarlet mixture. **C.** Cells transfected and analyzed as in **A**, but with 39 ng total AAV8/eGFP/mScarlet mixture. **D.** Cells transfected and analyzed as in **A**, but with 3.9 ng total AAV8/eGFP/mScarlet mixture. **E.** Cells transfected and analyzed as in **A**, but with 390 pg total AAV8/eGFP/mScarlet mixture.

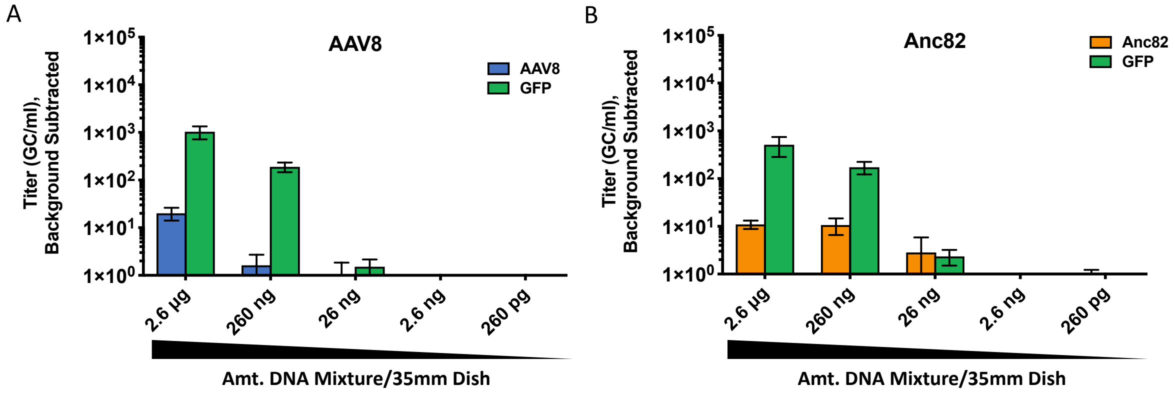

Figure S2: Effects of dilution on negative control 'libraries'. A. qPCR titration of AAV8/GFP 'libraries' created through transfection of dilutions of recombinant pAAVector.2/8 backbone along with GFP/luciferase. Titers of non-producing negative control preps were used to normalize values. Values represent average of three independent experiments, and error bars represent SEM. **B**. Same methods and computations as **A**, only using pAAVector.2/Anc82 backbone.

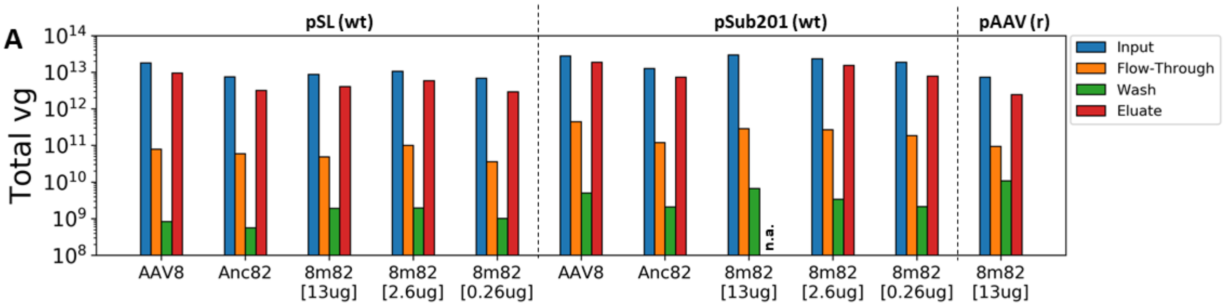

B

Yields (%)	AAV8	Anc82	AAV8m82 [13ug]	AAV8m82 [2.6ug]	AAV8m82 [0.26ug]
pSL-AAV	52.6	42.2	46.7	55.4	42.6
pSub201-AAV	68.2	58.5	n.a.	66.5	42.5
pAAV-AAV	-	-	33.2	-	-

Fig S3: Assessment of the bias of our library purification method. A. vg levels, quantified in the input, flow-through, wash and elution fraction by ITR2-free qPCR. **B**. vg recovery in the elution fractions.

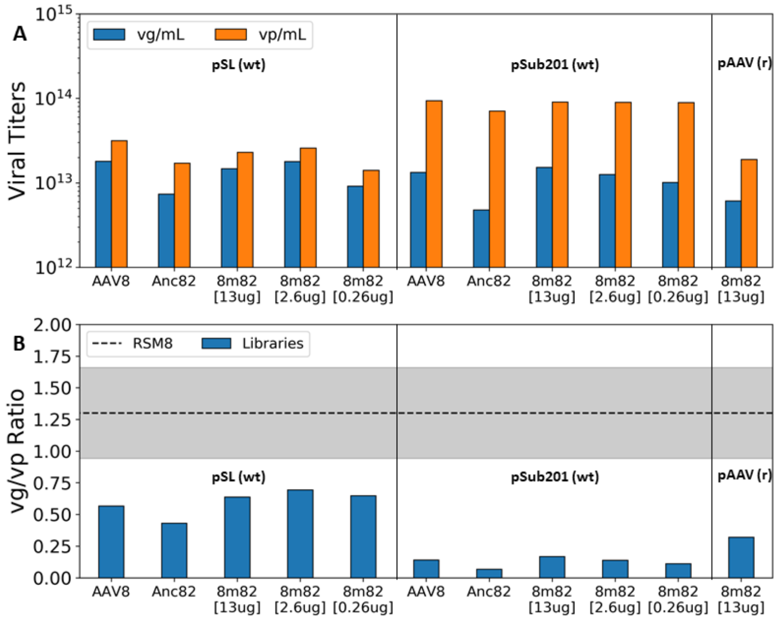

C

Preparation	%Full Capsids
RSM8	~100
pSL-AAV8	43.68
pSL-Anc82	33.24
pSL-AAV8m82 [13ug]	49.16
pSL-AAV8m82 [2.6ug]	53.37
pSL-AAV8m82 [0.26ug]	49.97
pSub201-AAV8	10.87
pSub201-Anc82	5.20
pSub201-AAV8m82 [13ug]	13.02
pSub201-AAV8m82 [2.6ug]	10.78
pSub201-AAV8m82 [0.26ug]	8.68
pAAV-AAV8m82 [13ug]	24.66

Fig S4: Quantification of vg levels, vp levels, and full/empty particle ratios in concentrated, buffer-exchanged AAV library preparations. A. vg and vp titers, measured by ITR2-free qPCR and SDS- PAGE densitometry, respectively. **B**. vg/vp ratios calculated for every library preparation. The mean experimental ratio obtained for the RSM8 (~100% full particles) is represented in dashed line. The shaded gray area represents the standard deviation from the mean (n=3 independent qPCR and ELISA assays). **C**. Assessment of the fraction of full particles in AAV library preparations.

Well	Preparation
1	pSL-AAV8
2	pSL-Anc82
3	pSL-AAV8m82 [13ug]
4	pSL-AAV8m82 [2.6ug]
5	pSL-AAV8m82 [0.26ug]
6	pSub201-AAV8
7	pSub201-Anc82
8	pSub201-AAV8m82 [13ug]
9	pSub201-AAV8m82 [2.6ug]
10	pSub201-AAV8m82 [0.26ug]
11	pAAV-AAV8m82 [13ug]

A

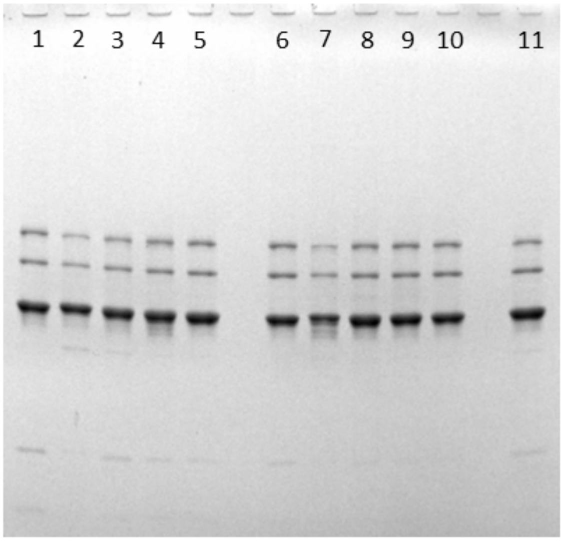

B

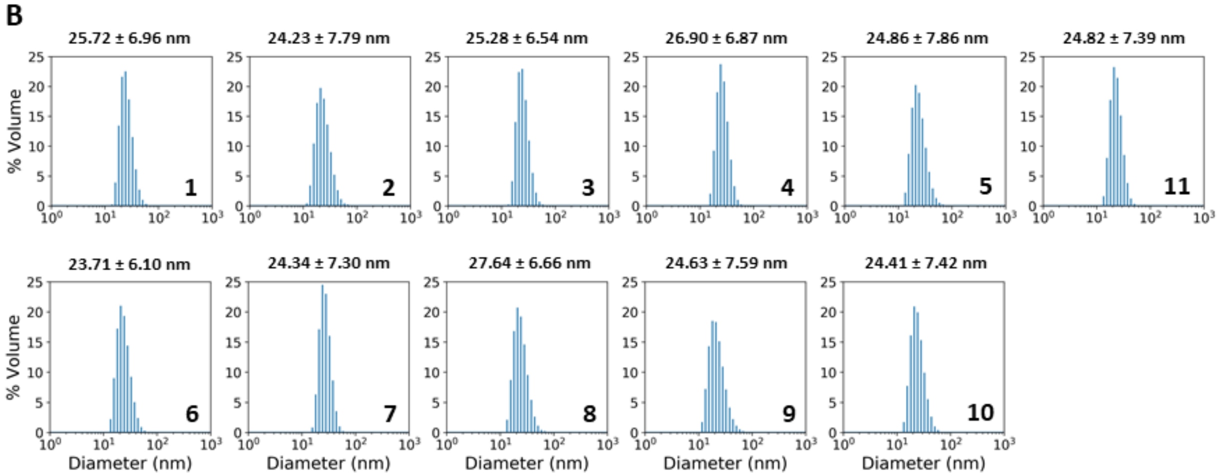

Fig S5: Evaluation of AAV library purity and size homogeneity. **A**. SDS-PAGE analysis of AAV library purity, at $1.4x10^{11}$ vp/well (Coomassie blue staining). **B**. Dynamic Light Scattering (DLS) analysis of AAV library size homogeneity.

.

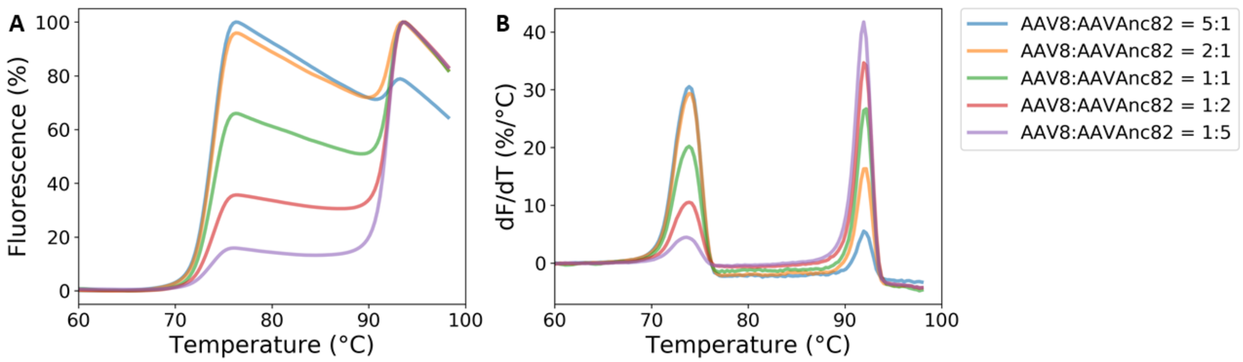

Fig S6. Impact of AAV8 and Anc82 relative vp levels on DSF. Normalized fluorescence (**A**) and fluorescence derivative (**B**) obtained at various AAV8:Anc82 stoichiometries. Preparations were all analyzed at 7.7x10^{11} vp/well.

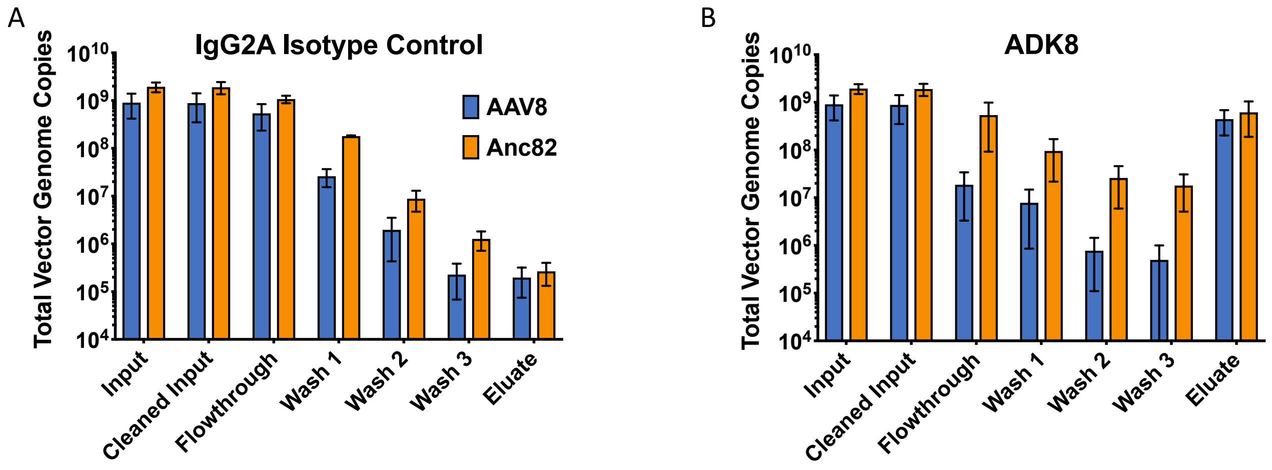

Figure S7: Immunoprecipitation of AAV8 and Anc82 particles. A. Pulldowns of AAV8 or Anc82 particles with a mouse IgG2A Isotype control antibody. Crude preps were applied to antibody coated Protein A/G agarose beads. Fractions were taken at each stage and titered using AAV8 or Anc82 capsid specific Taqman probes. Error bars represent SEM of three independent experiments. **B.** Pulldowns of AAV8 or Anc82 particles with a ADK8. Methods and computations are identical to **A.**

Figure S8: Derivation of mathematical model describing packaging and mosaicism

For each vector population analyzed, there is cDNA for 2 samples (ADK8 treated and isotype treated). Each of these cDNA samples is measured by RT-qPCR for AAV8 capsid, Anc82 capsid, and beta-actin. To calculate r_{total} for a given genome species, 4 values are required:

Isotype Treated cDNA	ADK8 Treated cDNA
$Ct_{capsid, isotype}$	$Ct_{capsid,ADK8}$
$Ct_{actin, isotype}$	$Ct_{actin,ADK8}$

Calculate dCt values to normalize for cDNA quality:

$$\Delta Ct_{isotype} = Ct_{capsid,isotype} - Ct_{actin,isotype} \tag{1}$$

$$\Delta Ct_{ADK8} = Ct_{capsid,ADK8} - Ct_{actin,ADK8} \tag{2}$$

Calculate ddCt value:

$$\Delta\Delta Ct = \Delta Ct_{ADK8} - \Delta Ct_{isotype} \tag{3}$$

Convert to fold change:

$$\text{Fold Change} = 2^{-\Delta\Delta Ct} = r_{total} \tag{4}$$

Where r_{total} is the fold change in cDNA abundance with ADK8 treatment, otherwise known as the **neutralization coefficient** of the vector population

$$r_{total} = \frac{\text{cDNA following ADK8 treatment}}{\text{cDNA following isotype control treatment}} = \frac{Y_{total}}{X_{total}} \tag{5}$$

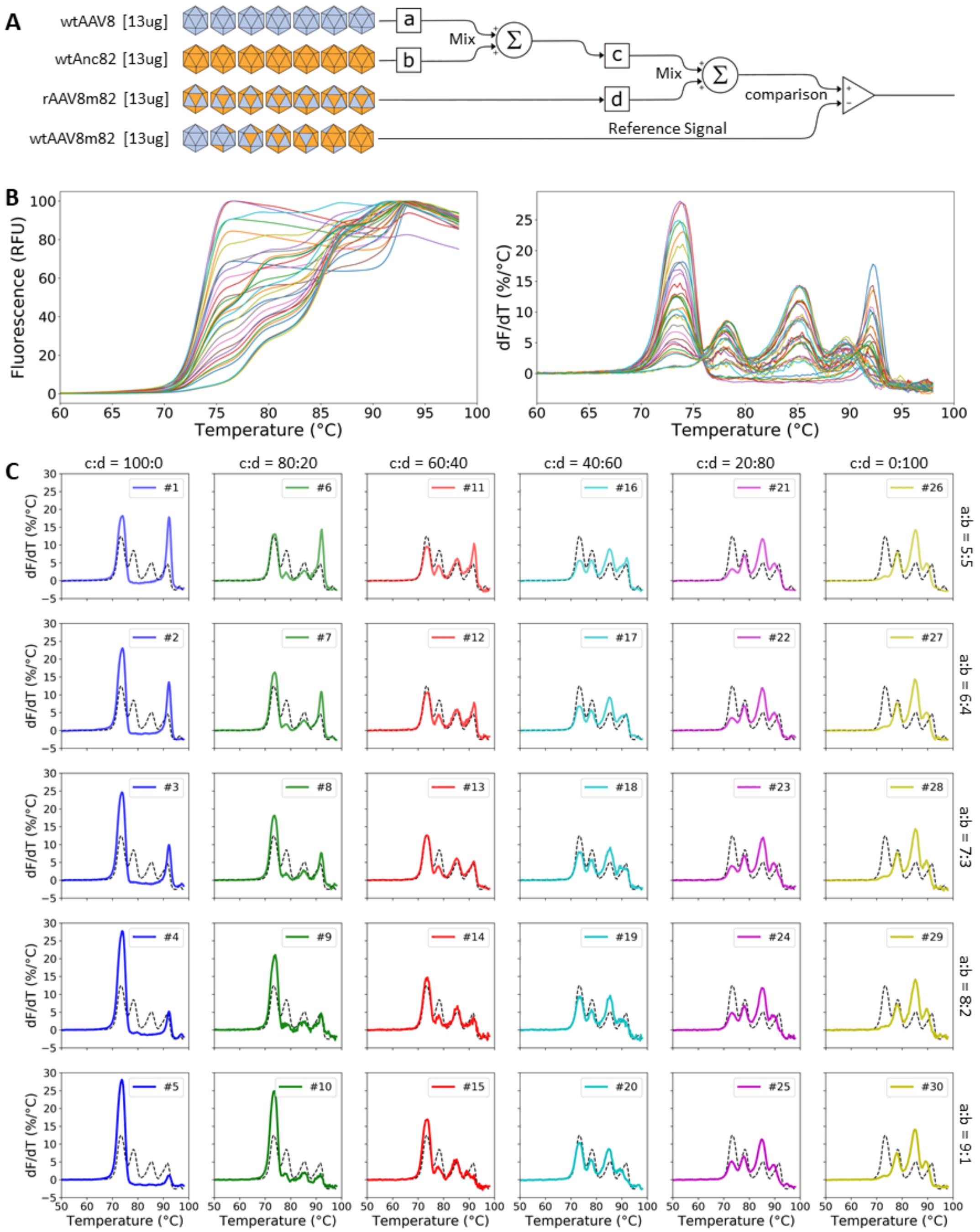

Fig S9. Recapitulation of pSub201-AAV8m82 [13ug] fluorescence signal through analysis of a mix of pSub201-AAV8, pSub201-Anc82 and rAAV8m82 [13ug] particles. **A**. Graphical representation of the different wtAAV8:wtAnc82:rAAV8m82 stoichiometries tested to recapitulate wtAAV8m82 fluorescence signal. The ratio a:b varied between 5:5 and 9:1, based on our previous observation that AAV8 produced better than Anc82. The ratio c:d varied between 100:0 and 0:100. (**B**, **C**). Fluorescence and derivative fluorescence signals obtained for the entire set of preparations, analyzed at $3x10^{11}$ vp/well. **D**. Individual derivative fluorescence signals, overlapped with the reference signal, obtained for pSub201-AAV8m82[13ug] (dashed line).

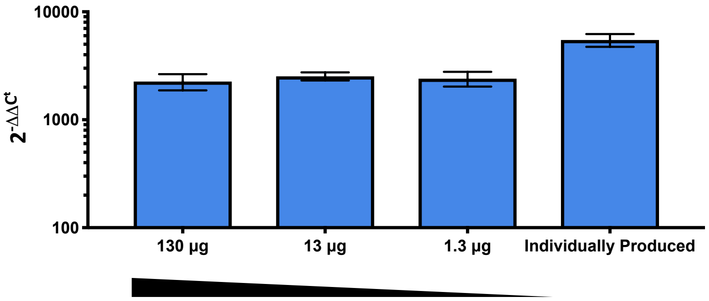

Figure S10: Relative abundance of transgene DNA in liver gDNA of mice injected with barcoded libraries. Liver gDNA of injected mice was probed for Tfrc and CMV DNA sequences to measure relative abundance of transgene DNA in livers.

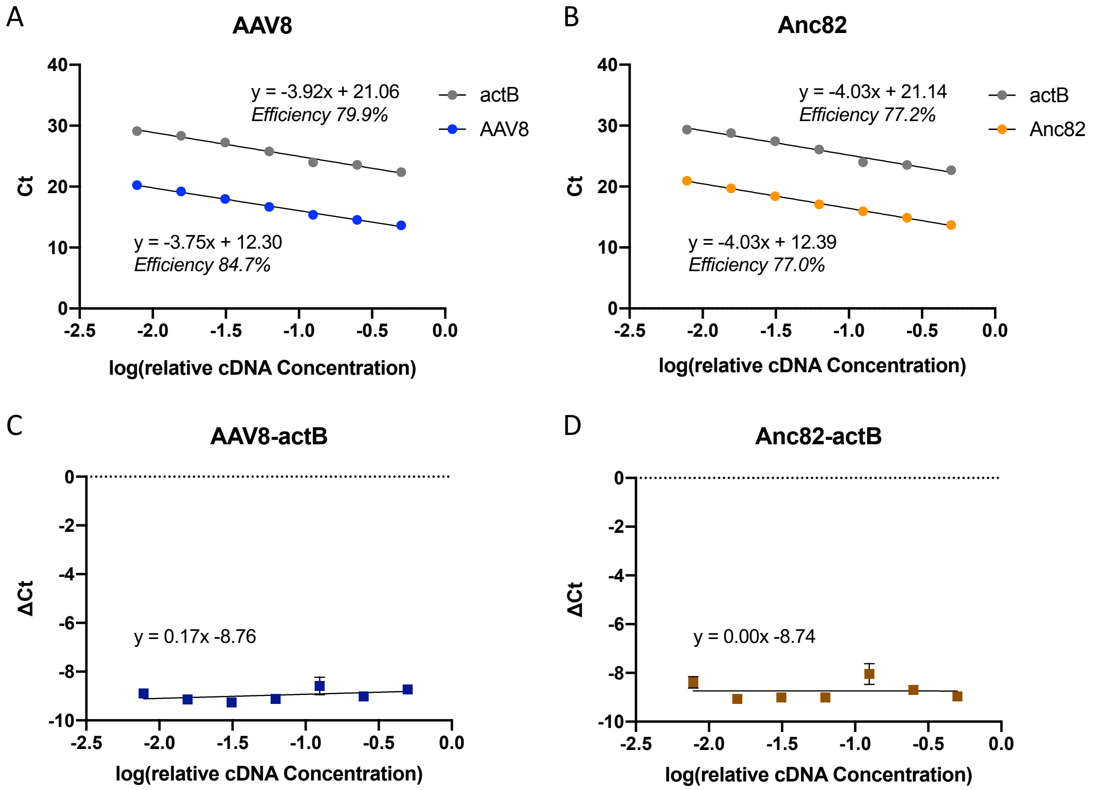

Figure S11: Efficiency of Taqman probes used in RT-qPCR. A. Relative quantification of beta actin and AAV8 capsid in serial dilutions of cDNA from HEK293 cells transfected with pAAVector2/8 using PEI. Assay efficiency is determined by the slope of the line. **B.** Similar to **A**, but using cDNA from cells transfected with pAAVector2/Anc82. **C.** Delta Ct calculations using data from **A** across cDNA concentrations. **D.** Delta Ct calculations using data from **B** across cDNA concentrations.

Table S1. **Summary of the melting temperatures** obtained for the SL (top) and pSub201 (bottom) AAV library preparations. (n=3 independent runs).

A

pSL-AAV	AAV8+Anc82	AAV8m82 [13ug]	AAV8m82 [2.6ug]	AAV8m82 [0.26ug]
Tm1 (°C)	73.37 ± 0.15	73.2 ± 0.0	73.46 ± 0.0	73.64 ± 0.15
Tm2 (°C)	-	77.98 ± 0.15	78.48 ± 0.58	77.98 ± 0.15
Tm3 (°C)	-	85.19 ± 0.0	85.36 ± 0.30	84.93 ± 0.26
Tm4 (°C)	91.79 ± 0.15	91.19 ± 0.0	91.79 ± 0.15	91.96 ± 0.0

B

pSub201-AAV	AAV8+Anc82	AAV8m82 [13ug]	AAV8m82 [2.6ug]	AAV8m82 [0.26ug]
Tm1 (°C)	73.46 ± 0.0	73.11 ± 0.15	73.37 ± 0.15	73.46 ± 0.0
Tm2 (°C)	-	77.89 ± 0.0	77.98 ± 0.15	78.07 ± 0.31
Tm3 (°C)	-	84.84 ± 0.15	85.10 ± 0.54	84.50 ± 0.65
Tm4 (°C)	91.61 ± 0.15	91.18 ± 0.45	91.44 ± 0.0	91.61 ± 0.15

Table S2: Oligonucleotides Used for Cloning

Primer	Orientation	Sequence
**KpnI-r2cAnc82**	Forward	5’-cagacaggtaccaaaacaaatgttctcg-3’
**r2cAnc82-AgeI**	Reverse	5’-gaattaaccggtttattgattaacaagcaaactagtttacagattacgg-3’
**HindIII-r2c8-t2a-mCherry**	Forward	5’-gacgcggaagcttcgatcaactac-3’
**r2c8-t2a-mCherry-SpeI**	Reverse	5’-taagcaactagtctacttgtacagctcgtccatgccgc-3’

Table S3: Transfection Mixes for Production of AAV8/Anc82 Capsid Libraries for DSF

System	Transfection Mix (for one 15-cm dish; X = 13ug, 2.6 ug, 1.3ug, 260 ng or 130 ng)
**pSub201**	26 ug ΔF6 Helper + X/2 ug pSub201-r2c8 + X/2 ug pSub201-r2cAnc82 + (26-X) ug pSEAP2
**pSL**	26 ug ΔF6 Helper + X/2 ug pSL-r2c8 + X/2 ug pSL-r2cAnc82 + 13 ug pRep2 + (13-X) ug pSEAP2
**pAAV**	26 ug ΔF6 Helper + 6.5 ug pAAV-r2c8 + 6.5 ug pAAV-r2cAnc82 + 13 ug pTransgene

Table S4: Transfection Mixes for Production of AAV8/Anc82 Capsid Libraries for Cross-packaging Analysis. For the sake of consistency with DSF libraries, conditions are labeled by the amount of total pAAV plasmid that would be transfected in a recombinant AAV production. Since the sizes of pSL and pSub201 constructs vary, the actual quantities of each plasmid in the mixture were adjusted to ensure equimolar *cap* amounts across conditions, and pDummy plasmid was added to normalize the total ug quantity of DNA transfected

System	ΔF6 Helper	AAV8 Plasmid	Anc82 Plasmid	pRep	pDummy (promoter free plasmid)
**pSL – 13 ug**	26 ug	5.7 ug	5.7 ug	9.91 ug	
**pSL – 1.3 ug**	26 ug	570 ng	570 ng	9.91 ug	10.3 ug
**pSL – 130 ng**	26 ug	57 ng	57 ng	9.91 ug	11.3 ug
**pSub201 – 13 ug**	26 ug	7.4 ug	7.4 ug		6.6 ug
**pSub201 – 1.3 ug**	26 ug	740 ng	740 ng		19.8 ug
**pSub201 – 130 ng**	26 ug	74 ng	74 ng		21.2 ug

Table S5: Oligonucleotides Used for qPCR

	Forward	Reverse	Probe
CMV2	5'-CATCTACGTATTAGTCATCGCTATTACCA-3'	5'-GAAATCCCCGTGAGTCAAACC-3'	5'-TCAATGGGCGTGGATAG-3'
eGFP	5'-AGCAAAGACCCCAACGAGAA-3'	5'-GGCGGCGGTCACGAA-3'	5'-CGCGATCACATGGTCCTGCTGG-3'
AAV8 Capsid	5'-TTTGCCTGGACTGCTGGG-3'	5'-TGCCAAAAATCAGGATCCCGTTAC-3'	5'-AAGAAATTCATTGGCTAATCCTGGCATCG-3'
Anc82 Capsid	5'-CTACGGGAGGCACAGCGG-3'	5'-GTCCAGGCAGCCAGTTTTTG-3'	5'-CCAGACGTTGCAGTTTTCTCAGGCC-3'
Hs Actin	5'-ACAGAGCCTCGCCTTTG-3'	5'-CCTTGCACATGCCGGAG-3'	5'-TCATCCATGGTGAGCTGGCGG-3'
Library BC Amplification	5'-TCGTCGGCAGCGTCAGATGTGTATAAGAGACAGGAGGTCTATATAAGCAGAGCTGGTTTAGTGAACCGT-3'	5'-GTCTCGTGGGCTCGGAGATGTGTATAAGAGACAGGACGAGAACATTTGTTTTGGTACCTGTCTGCGTAG-3'	

Supplemental Methods:

FACS: Individual wells of 6 well plates containing HEK293 cells at approximately 90% confluency were transfected with a total of 6.5 µg DNA using PEI Max, including 2.6 µg deltaF6, between 3.9 µg-390 pg of an equimolar mixture of pAAVector2/8, ITR-CMV.eGFP plasmid, and ITR-CMV.mScarlet, and a dummy plasmid to control for total DNA added. Cells were harvested after 48 hours by trypsinization, washed with FACS buffer (1X DPBS (Corning) supplemented with 5% fetal bovine serum and 2% EDTA), and resuspended in FACS buffer before being subjected to flow cytometry.

SDS-PAGE Densitometry: For quantification of vp levels, SDS-PAGE were run as described in methods. STR8, analyzed three times by ELISA using the RSM8, was used to generate a standard curve. STR8 was first subjected to 1.5-fold serial dilution, between 3.32×10^{13} pt/mL (1:1) and 4.37×10^{12} pt/mL (1:7.59375). 10 µL of each standard were mixed with 5 µL PBD2+ and 5 µL 1X NuPAGE lithium dodecyl sulfate (LDS) sample buffer (NP007, ThermoFisher) (Boston) or 1X Laemmli sample buffer (161-0747, Biorad) (Nantes). Diluted unknown samples (dilution factors determined empirically) were subjected to the same treatment. 20 µL SDS-PAGE mixes were incubated for 10 min at 99 °C and subjected to a quick spin. 15 µL of each mix were further loaded on a 10-well polyacrylamide gel (see above for the references of the gels used in Nantes and Boston), and run for 2.5-3 h at 100 V. Gels were stained as described above and imaged. For the standard and unknown samples, the pixel density of VP3 was measured using Fiji. The standard curve was further fitted using a second order polynomial function, used to calculate the vp levels in each unknown sample based on their VP3 pixel density value.

Immunoprecipitations: Crude preps of vector produced from pSL-AAV8 or pSL-Anc82 were diluted to 7.50E+09 GC/ml and treated with either mouse IgG2A kappa or ADK8 antibody at a final dilution of 1:1250. Immunoprecipitations were preformed using Pierce™ Protein A/G Agarose (20423, Thermo Fisher). Vector Particles were eluted in 0.2 M glycine, pH 2.8 and neutralized in equal volumes of 100 mM Tris pH 8.5. All fractions were collected, and DNAse I-protected total vector genomes were quantified using TaqMan probes against AAV8 or Anc82 and the Applied Biosystems 7500 Real-time PCR System.

Liver Transgene Abundance Assay: Liver gDNA isolated from injected mice was quantified using a TaqMan™ Tfrc Copy Number Reference Assay (4458366, Thermo Fisher) and a TaqMan assay recognizing CMV2 promoter DNA sequence. 25 ng total gDNA was measured per 25 µl reaction. qPCR was carried out using the Applied Biosystems 7500 Real-time PCR System.

TaqMan Probe Efficiency Assay: Individual wells of 6 well plates containing HEK293 cells at approximately 90% confluency were transfected with 2.6 µg deltaF6 and with either 1.3 µg pAAVector2/8 or 1.3 µg pAAVector2/82 using PEI Max. Cells were harvested in TriZol and RNA was extracted and cDNA synthesized as described in methods. Serial dilutions of cDNA were used as input for RT-qPCR reactions.
