# Supplementary material for: Cross-Packaging and Capsid Mosaic Formation in Multiplexed AAV Libraries
Source: Mol Ther Methods Clin Dev. 2019 Nov 26;17:107–21. doi: 10.1016/j.omtm.2019.11.014 (PMC6938944; doi:10.1016/j.omtm.2019.11.014)
Supplement: Document S2. Article plus Supplemental Information [file mmc2.pdf]

Molecular Therapy
Methods & Clinical Development
Original Article

Cross-Packaging and Capsid Mosaic Formation in Multiplexed AAV Libraries

Pauline F. Schmit,[1,6] Simon Pacouret,[1,5,6] Eric Zinn,[1] Elizabeth Telford,[1] Fotini Nicolaou,[1] Frédéric Broucque,[5] Eva Andres-Mateos,[1] Ru Xiao,[1] Magalie Penaud-Budloo,[5] Mohammed Bouzelha,[5] Nicolas Jaulin,[5] Oumeya Adjali,[5] Eduard Ayuso,[5] and Luk H. Vandenberghe[1,2,3,4]

[1]Grousbeck Gene Therapy Center, Schepens Eye Research Institute, Mass Eye and Ear, Boston, MA 02114, USA; [2]Ocular Genomics Institute, Department of Ophthalmology, Harvard Medical School, Boston, MA 02114, USA; [3]The Broad Institute of Harvard and MIT, Cambridge, MA, USA; [4]Harvard Stem Cell Institute, Harvard University, Cambridge, MA, USA; [5]INSERM UMR1089, University of Nantes, Centre Hospitalier Universitaire, Nantes, France

Generation and screening of libraries of adeno-associated virus (AAV) variants have emerged as a powerful method for identifying novel capsids for gene therapy applications. For the majority of libraries, vast population diversity requires multiplexed production, in which a library of inverted terminal repeat (ITR)-containing plasmid variants is transfected together into cells to generate the viral library. This process has the potential to be confounded by cross-packaging and mosaicism, in which particles are comprised of genomes and capsid monomers derived from different library members. Here, we investigate the prevalence of cross-packaging and mosaicism in simplified, minimal libraries using novel assays designed to assess capsid composition and packaging fidelity. We show that AAV library variants are prone to cross-packaging and capsid mosaic formation when produced at high plasmid levels, although to a lesser extent than in a recombinant context. We also provide experimental evidence that dilution of input library DNA significantly increases capsid monomer homogeneity and increases capsid:genome correlation in AAV libraries. Lastly, we determine that similar dilution methods yield higher-quality libraries when used for *in vivo* screens. Together, these findings quantitatively characterized the prevalence of cross-packaging and mosaicism in AAV libraries and established conditions that minimize related noise in subsequent screens.

INTRODUCTION

The adeno-associated virus (AAV) is a 20- to 25-nm, nonenveloped, single-stranded DNA (ssDNA) virus that belongs to the family Parvoviridae. Its 4.7-kb genome, comprised of *rep* and *cap* genes, is packaged into a T = 1 icosahedral capsid composed of 60 C-terminally overlapping protein subunits encoded by *cap*, VP1, VP2, and VP3, present at the ratio 1:1:10.[1] Inverted terminal repeats (ITRs) play a key role in genome replication and DNA packaging and constitute the only *cis*-acting elements required for genome encapsidation.[2] Due to its low immunogenicity, nonpathogenicity, and ability to mediate long-term transgene expression in both dividing and nondividing cells,[3] AAV has emerged as a preferred platform for gene therapy.[4] Yet, the success of AAV-based therapeutic strategies is limited by pre-existing immunity,[5] the nonspecific tropism of AAV vectors,[6] and inefficient production hampering manufacturing vectors at therapeutic scale.[7] To address these challenges, the scientific community is currently investing major efforts in the engineering of novel AAV capsids, capable of evading neutralizing antibodies (NAbs), while efficiently producing and maintaining specific tissue tropism.

AAV libraries have emerged a powerful tool for AAV capsid engineering. Viral libraries can be produced by transfection of HEK293 cells with pools of ITR-containing *rep/cap* plasmid variants, purified and subjected to a selective screen. Selected variants can be vectorized and further characterized in terms of production, tissue tropism, and antibody neutralization. Such strategies have yielded numerous promising synthetic capsid variants that outperform their natural counterparts, including the liver-tropic AAVDJ,[8] the muscle-tropic AAVMYO,[9] and AAV7m8, a variant capable of photoreceptor transduction upon intravitreal vector administration.[10] More recently, efforts have focused on high throughput, systematic assessment of library variant phenotypes, through Illumina next-generation sequencing (NGS) analysis of barcoded AAVs.[11] Results from these experiments can be used to draw high-resolution, sequence-function heatmaps, which can then be used as blueprints to design improved gene delivery vectors that address specific therapeutic needs.

However, these strategies are only valid in the absence of the following: (1) genome cross-packaging among capsid isolates, whereby an AAV genome is packaged into a mismatched capsid, and (2) capsid mosaic formation, a phenomenon defined by oligomerization of distinct variant VP1-3 proteins. To limit cross-packaging and mosaicism in AAV library preparations (preps), the current

Received 18 August 2019; accepted 5 November 2019;
https://doi.org/10.1016/j.omtm.2019.11.014.

[6]These authors contributed equally to this work.

Correspondence: Luk H. Vandenberghe, Grousbeck Gene Therapy Center, Schepens Eye Research Institute, Mass Eye and Ear, Boston, MA 02114, USA.
E-mail: luk_vandenberghe@meei.harvard.edu
Correspondence: Eduard Ayuso, INSERM UMR1089, University of Nantes, Centre Hospitalier Universitaire, Nantes, France.
E-mail: eduard.ayuso@univ-nantes.fr

This is an open access article under the CC BY license (http://creativecommons.org/licenses/by/4.0/).

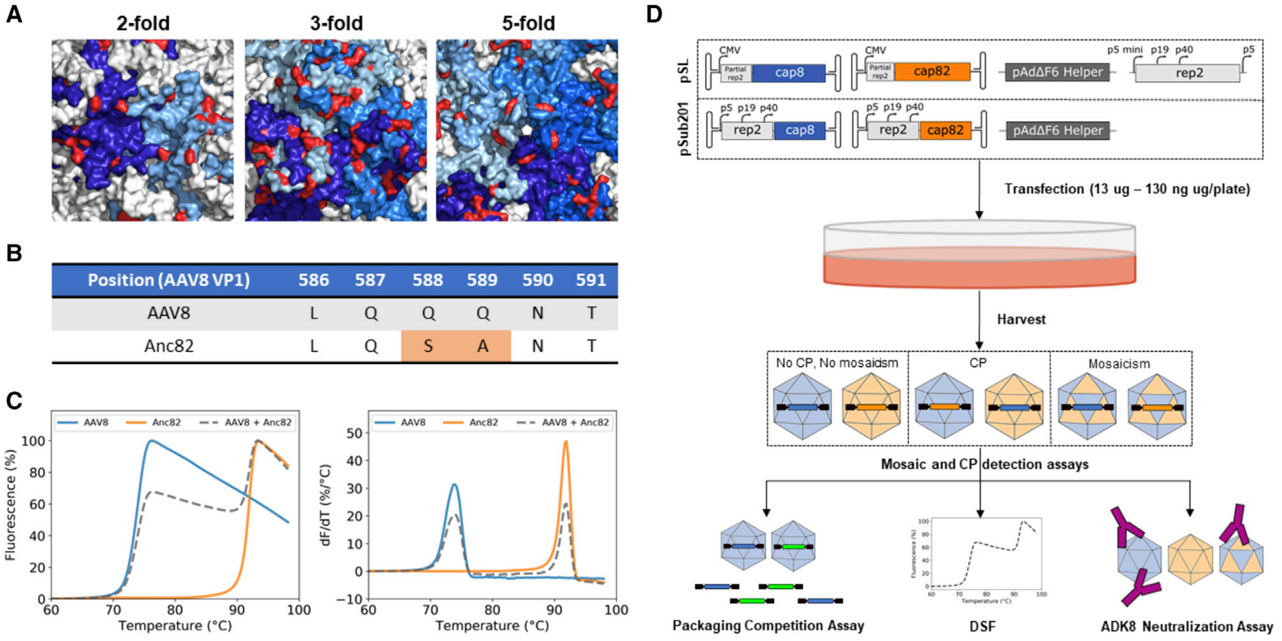

Figure 1. Presentation of the AAV8/Anc82 Experimental Design Used to Study Capsid Mosaic Formation and Cross-Packaging

(A) Sequence variability represented at the surface of the AAV8 capsid (PDB: 2QA0). Variable residues between AAV8 and Anc82 are represented in red. (B) AAV8 and Anc82 VP1 sequence alignment between amino acid positions 586 and 591. AAV8 capsid-specific ADK8 antibodies are known to bind this motif, disrupted in the case of Anc82. (C) Thermal stability profiles of AAV8 (blue), Anc82 (orange), and a mix of both particles at a 1:1 vp ratio (gray, dashed line). Left: normalized fluorescence signals; right: derivative fluorescence signals. (D) Overview on the experimental procedure. AAV8/Anc82 libraries were produced through cotransfection of HEK293 cells with a decreasing amount of *rep2/cap8* and *rep2/capAnc82* plasmids, using either the pSL or pSub201 production system. AAVs were further harvested and subjected to a battery of assays to investigate cross-packaging and capsid mosaic formation.

standard method consists of transfecting producing cells with very low levels of plasmid library, down to 10 ng per 15-cm dish.[12,13] In theory, this approach should favor the internalization of a single *rep/cap* plasmid per cell, hence decreasing the probability of cross-packaging and capsid mosaic formation. Recently, Nonnenmacher et al.[14] provided compelling evidence that capsid mosaic formation and cross-packaging were limited in a wild-type (WT) production context, hence facilitating the generation of AAV libraries for capsid engineering and sequence-function studies.

Here, we aim to characterize and quantify how production conditions influence the abundance of cross-packaging and capsid mosaicism in order to understand better the process of library production and establish a protocol that maximizes the titer-to-noise ratio in library screens. Minimal AAV libraries composed of two distinct capsid variants were produced through cotransfection of HEK293 cells with decreasing levels of *rep/cap* plasmids, encoding AAV8,[15] a natural serotype, and Anc82,[16] a putative ancestral capsid sharing 94.7% of its sequence identity with AAV8. Virus pools were harvested and subjected to a battery of characterization assays, allowing us to investigate cross-packaging, capsid mosaic formation, as well as the intricate relationship between both phenomena. In this study, we show that cross-packaging and mosaicism are prominent in AAV8/Anc82 libraries produced in saturation conditions (13 μg *rep/cap* plasmid per 15-cm plate), and we provide experimental evidence that this phenomena can be attenuated by lowering the dose of *rep/cap* plasmids used for cotransfection. Our results also indicate that production of AAV8/Anc82 libraries at high plasmid levels results in capsid mosaics with high VP composition heterogeneity, with a tendency toward VP-genome correlation. In addition, in line with Nonnenmacher et al.,[14] we find that the presence or absence of ITRs in *rep/cap* production plasmids influences capsid mosaic homogeneity in terms of VP stoichiometry. Lastly, we extend our study to more complex AAV libraries and provide optimized experimental conditions to limit cross-packaging and mosaicism without compromising viral titers.

RESULTS

Experimental Model, Cross-Packaging, and Mosaicism Assays

In order to study cross-packaging and mosaicism in AAV library preps, we chose a simple n = 2 library model based on AAV8 and Anc82, a computationally predicted ancestor of AAV8. These two serotypes were chosen based on three criteria. First, AAV8 and Anc82 exhibited a VP sequence homology of 94.7%. According to the literature, this relatively high sequence similarity was likely to be sufficient to permit oligomerization of VPs from both serotypes, i.e., capsid mosaic formation[17] (Figure 1A). Second, the antigenic epitope of the anti-AAV8 monoclonal NAb ADK8[18] was shown to be disrupted in the Anc82 capsid (Figure 1B), whereas both serotypes exhibited very different capsid melting temperatures (Tm^{AAV8} = 73.87 ± 0.13°C; Tm^{Anc82} = 91.95 ± 0.01°C) (Figure 1C). These structural

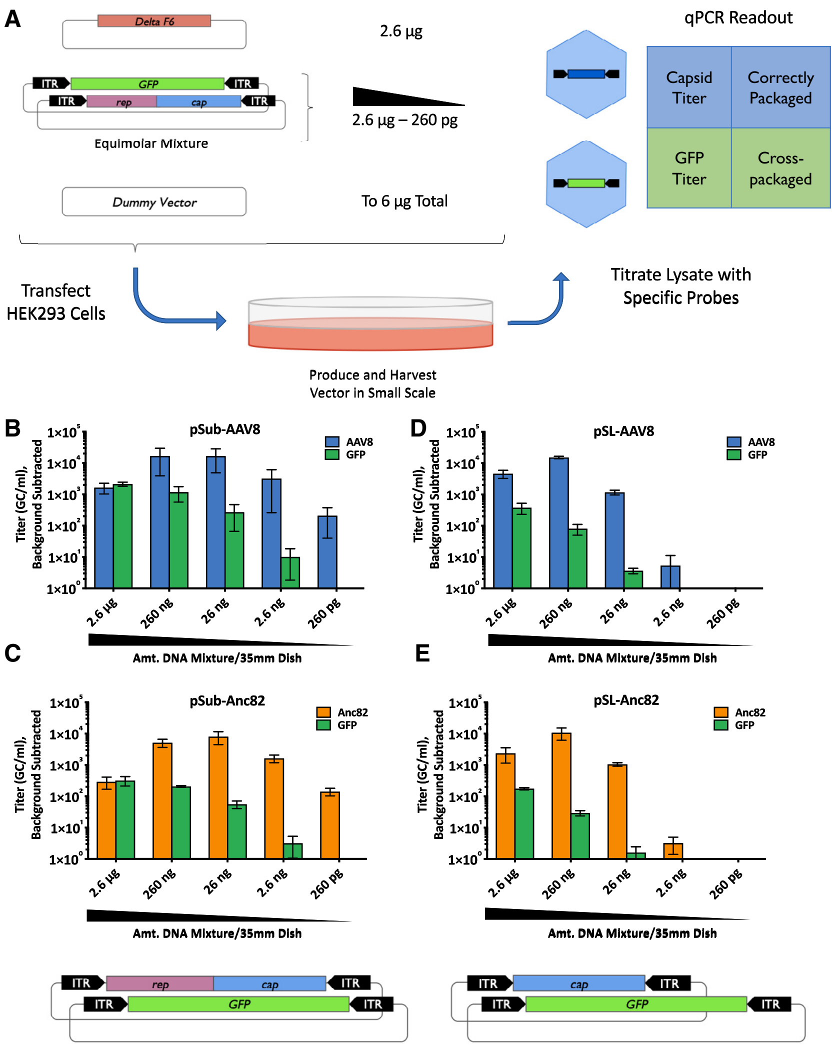

Figure 2. Dilution of Input Library Reduces Incidence of Cross-Packaging

(A) Schematic of experimental design. Plasmids were mixed as indicated and transfected into HEK293 cells using PEI Max. Crude viral preparations (preps) were harvested 72 h post-transfection and DNase-protected vector genomes titered by qPCR using AAV capsid and GFPspecific TaqMan probes. (B) qPCR titration of AAV8/GFP-luciferase "libraries" created through transfection of dilutions of WT pSub201.AAV8 backbone along with GFP-luciferase. Titers of nonproducing negative control preps were used to normalize values. Values represent the average of three independent experiments, and error bars represent SEM. (C) qPCR titration of libraries produced using a mixture pSub201.Anc82 and GFP GFP-luciferase plasmids. An Anc82-specific TaqMan probe was used for titration, and data were generated and analyzed as above. (D) qPCR titration of SL-AAV8/GFP-luciferase libraries generated exactly as above but including a P5-driven *rep* plasmid provided in *trans* to ensure equimolar *rep:cap* ratios in the mixes. Data were generated and analyzed as above. (E) qPCR titration of libraries produced using a mixture of pSL-Anc82 and GFP-luciferase plasmids. Data were generated and analyzed as above.

differences were a key element in this study, allowing us to discriminate among AAV8, Anc82, and mosaic capsids, based on their interactions with ADK8 and thermal stability. Lastly, previous studies showed that AAV8 and Anc82 produced at similarly high titers.[16] This was an important consideration to ensure that both serotypes could be produced at titers compatible with our cross-packaging and capsid mosaic detection assays.

In this study, two library production plasmid systems were investigated (Figure 1D). In the first system, the *cap8* or *capAnc82* genes were cloned into the pSL plasmid, which includes an ITR-flanked AAV *cap* gene under the control of a cytomegalovirus (CMV) promoter (Figure 1D; pSL system). During production, *rep2* was supplemented in *trans* and was expressed under the control of a mini-p5 promoter (Figure 1D; pSL system). Such a system is relevant to the identification of functional capsid variants within AAV libraries, as it allows production of replication-defective AAVs, packaging a genome from which *cap* can be transcribed in most target tissues. In the second system, *cap8* and *capAnc82* were cloned into the pSub201 plasmid,[19] which is derived from a WTAAV2 and includes *rep* and *cap* genes under control of their native viral promoters (Figure 1D; pSub201 system). The pSub201 system was chosen based on the work of Nonnenmacher et al.,[14] who provided compelling evidence that cross-packaging and mosaicism were limited in this production context.

Following production in HEK293 cells, AAV library preps were subjected to a series of assays, allowing us to probe cross-packaging and capsid mosaic formation (Figure 1D). Both phenomena were first investigated independently by the means of a capsid/GFP packaging competition assay and a thermostability assay.[20] The link between cross-packaging and capsid mosaic formation was then studied by subjecting our AAV8/Anc82 libraries to a qRT-PCR/ADK8 neutralization assay with a serotype-specific readout (Figure 1D).

Cross-Packaging in Single Capsid Libraries

To address initially the question of cross-packaging, we first interrogated a simple system composed of only one capsid species. First, equimolar amounts of pSub201-AAV8 (Figure 1D) and an ITR-flanked GFP-luciferase plasmid were combined and transfected into HEK293 cells at 10-fold dilutions alongside equal quantities of ΔF6 adenovirus helper plasmids (Figure 2A). Total amounts of DNA transfected in each condition were kept constant with a promoter-free dummy plasmid. As negative controls, combinations of ITR-free *rep2/cap8* plasmids and GFP-luciferase transgene plasmids

were transfected at the same dilutions. Supernatants collected from these transfections were then titrated by TaqMan quantitative PCR (qPCR) using probes specific for each potential transgene.

We found that cross-packaging of GFP-luciferase transgenes was abundant at the highest input plasmid concentration and decreased dramatically upon dilution of this plasmid solution (Figure 2B). At a dilution approximating 1,000 plasmids per cell (26 ng condition), less than 2% of total packaged genomes contained GFP, the surrogate measure for cross-packaging. Flow cytometry data of cells transfected with decreasing quantities of an equimolar combination of two plasmids expressing EGFP and mScarlet showed that the number of transfected cells positive for both markers progressively decreased when lowering the amount of plasmid used for transfection (Figure S1). Surprisingly, titers of capsid-containing particles remained relatively high across dilutions, in contrast to negative-control transfections in which yields were nearly undetectable upon 100-fold dilution of the capsid and transgene plasmids (Figure S2). These findings were upheld in identical experiments with Anc82 plasmids (Figure 2C).

We then repeated these experiments with either pSL-AAV8 or pSL-Anc82, which do not contain *rep* in *cis* and drive *cap* expression from a CMV promoter rather than the viral p40 (Figure 1D). Cross-packaging of GFP-luciferase transgenes was even further diminished than with the WT backbones using both AAV8 and Anc82 plasmids (Figures 2D and 2E). However, capsid titers dropped more substantially at lower DNA inputs, suggesting that the native genome conformation offers some production benefit, either as a result of physical linkage of the *rep* and *cap* genes on the same ITR-containing DNA molecule, ensuring cotransfection and even stoichiometry of *rep* and *cap* copies in cells, or differences in AAV gene expression between the two systems. Overall, these data indicate that whereas cross-packaging is highly prevalent at maximum input DNA concentrations, packaging *cap*-containing genomes in dually transfected cells is increasingly abundant as input decreases.

Capsid Mosaic Formation in Minimal Two-Capsid Libraries

Next, we studied capsid mosaic formation in the context of multiplexed AAV library generation by the means of differential scanning fluorimetry (DSF). We reasoned that the absence of capsid mosaics would lead to DSF fingerprints resembling the clonal AAV8 and Anc82 particle profiles and that mosaicism would result in intermediate and aberrant DSF signals (Figure 1C).

A total of 11 AAV8/Anc82 libraries were produced, harvested, purified, and subjected to DSF. First, we verified that our affinity chromatography purification method, based on the POROS CaptureSelect AAVX Affinity Resin, was not overly biased toward one of the two serotypes under investigation and suitable for the purification of our AAV8/Anc82 libraries (Figure S3). With the aim of analyzing our AAV library preps by DSF at equal capsid concentration, we then quantified viral particle (vp) levels in every sample by SDS-PAGE densitometry (Figure S4A). DNaseI-resistant viral genome (vg) levels were also measured by ITR2-free qPCR[21] (Figure S4A). Both assays were calibrated using the Reference Standard Material serotype 8 (RSM8; ATCC VR-1816).[22] Last, we showed by SDS-PAGE and dynamic light scattering (DLS) that our purified preps were highly pure and homogeneous in size, suggesting that our DSF assay should be minimally prone to protein contaminant-related biases (Figure S5).

Following quality control, pSL and pSub201 AAV preps were subjected to DSF at 3.13E+11 vp/well. As expected, mixes of AAV8 and Anc82 particles, produced separately using the pSL and pSub201 systems (Figure 1D), yielded identical SYPRO Orange fluorescence fingerprints. These two reference signals exhibited two sharp fluorescence transitions, respectively, centered on 73°C and 92°C (Figures 3Ai and 3Aii; Table S1). Interestingly, the signals obtained for both the pSL and pSub201 AAV8/Anc82 libraries produced in saturation conditions (13 μg *rep/cap* per 15-cm dish) were highly similar, yet diverged from the reference signals (Figures 3Aiii and 3Aiv). In particular, the derivative signal exhibited four distinct peaks, centered on 73°C, 78°C, 85°C, and 91°C (Figure 3Aiv; Table S1). The two transitions occurring at 73°C and 91°C were reminiscent of those obtained for the mix of AAV8 and Anc82 particles (Figures 1C, 3Ai, and 3Aii), suggesting the existence of VP^{AAV8}:VP^{AAV8} and VP^{Anc82}:VP^{Anc82} interactions. However, with the consideration of the high purity and size homogeneity of the library preps (Figure S5), the transitions taking place at 78°C and 85°C were likely to indicate the existence VP^{AAV8}:VP^{Anc82} interactions within assembled AAV particles, i.e., the presence of capsid mosaics (Figure 3C).

As in our cross-packaging experiment (Figure 2), we investigated the impact of *rep/cap* plasmid dilution on capsid mosaic formation through DSF analysis of the AAV preps, which obtained cells with lower doses of *rep2/cap8* and *rep2/capAnc82* plasmids (2.6 μg or 0.26 μg total per 15-cm dish) at a 1:1 ratio (Figures 3Av–3Aviii). At 2.6 μg plasmid per plate (Figures 3Av and 3Avi), the amplitude of the fluorescence transitions occurring at 78°C and 85°C decreased relatively to those taking place at 73°C and 91°C. This phenomenon was even more pronounced at 0.26 μg plasmid per plate (Figures 3Avii and 3Aviii), a condition for which only two transitions could be observed. These results suggested that as expected, dilution of the two *rep/cap* plasmids used for library production resulted in a decrease in VP^{AAV8}:VP^{Anc82} interactions within assembled AAV particles, i.e., a decrease in capsid mosaic formation.

Nonetheless, the amplitude of Anc82 transition appeared to be lower than that of AAV8 in the signal obtained at low levels of *rep/cap* plasmids (Figures 3Av–3Aviii), particularly in the pSub201 production context. This is likely due to the fact that the AAV library, produced at 0.26 μg *rep/cap* per plate, contained fewer Anc82 than AAV8 capsids. Indeed, analysis by DSF of a set of AAV preps, obtained by mixing various ratios of separately produced pSL-AAV8 and pSL-Anc82 capsids, revealed that the relative amplitudes of both AAV8 and Anc82 transition correlated with the relative concentrations of these two species (Figure S6). This result was in line with the following

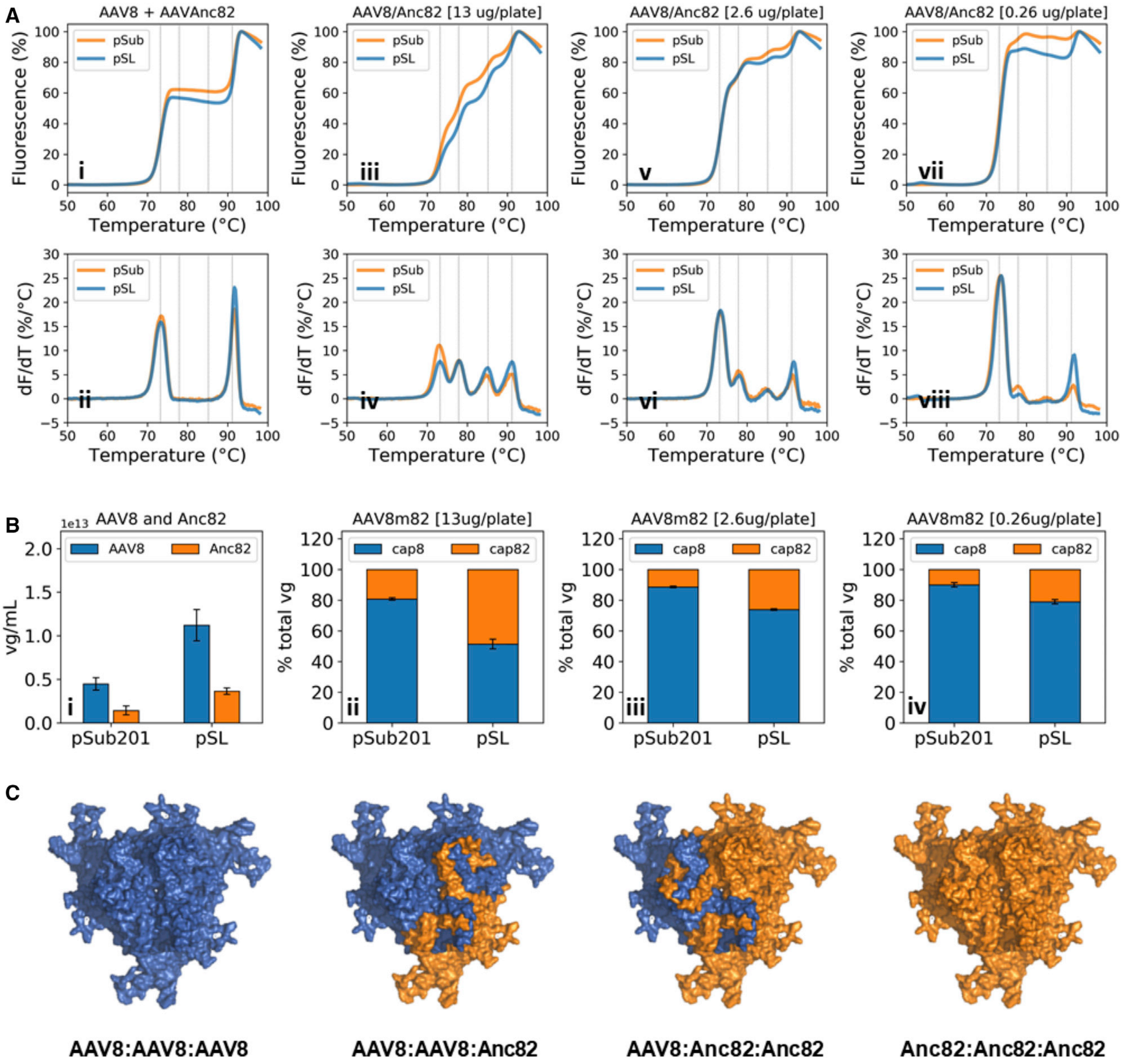

Figure 3. Study of Capsid Mosaic Formation in AAV8/Anc82 Library Preps

(A) Thermostability profiles of AAV8/Anc82 library preps produced with decreasing *rep/cap* plasmid levels. The AAV8 + Anc82 reference profile was obtained through analysis of a mix of equal amounts of AAV8 and Anc82 viral particles. All preps were analyzed at 3.13×10^{11} vp/well. (B) AAV8 and Anc82 vg distribution in AAV library preps. The titers represented on the left panel were obtained through quantification of AAV8 and Anc82 control preps that were produced and purified independently. Error bars represent the SD of the mean of independent experiments (n=3). (C) Representation of the four possible capsid trimer conformations in AAV8/Anc82 library preps.

facts: that (1) AAV8 produced slightly better than Anc82 (2.2- to 2.4-fold increase) (Figure S3A, blue bars) and (2) AAV8 purified with faintly higher yields than Anc82 (Figures S3B and S4A).

In order to determine whether AAV8 and Anc82 vp levels correlated with their respective vg levels, we subjected the ten preps to qPCR using primers and probes targeting *cap8* or *capAnc82*. In accordance with our ITR2-free qPCR titers (Figures S3A and S4A), vg levels were 3.1-fold higher in SL-AAV8 and pSub201-AAV8 than in SL-Anc82 and pSub201-Anc82 preps (Figure 3Bi). In addition, the relative amplitudes of the AAV8 (73°C) and Anc82 (91°C) Tm peaks correlated with the distribution of AAV8 and Anc82 DNaseI-resistant genomes in the different purified AAV libraries (Figures 3A and 3B). It is interesting to note that in the case of the AAV8/Anc82 prep produced in saturation condition with the pSL system (Figures 3Aiii, 3Aiv, and 3Bii), equal levels of *cap8* and *capAnc82* could be measured,

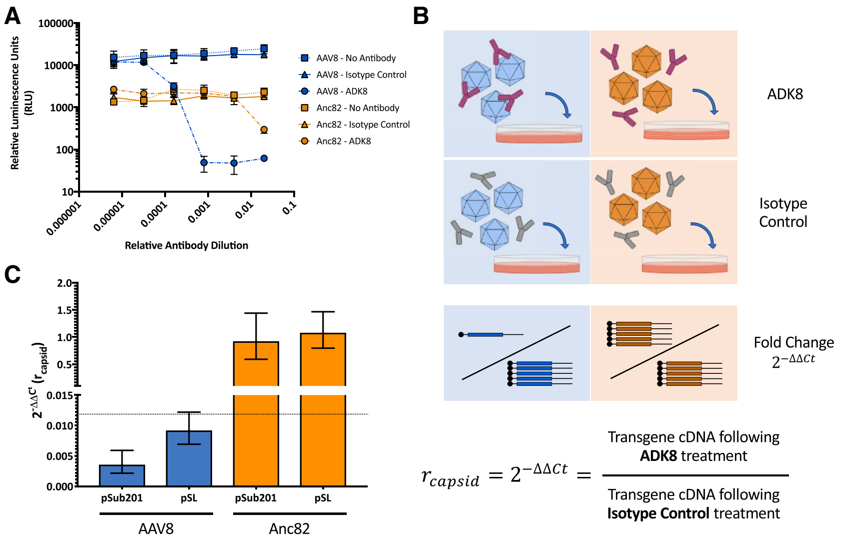

Figure 4. Monoclonal Antibody ADK8 Differentially Neutralizes AAV8 and Anc82 Capsids

(A) Neutralization of individually produced AAV8 and Anc82 prep by ADK8, as measured by luciferase assays of transfected HEK293 cells. Vectors were treated with ADK8, an IgG isotype control antibody, or no antibody. Starting concentrations of each antibody were measured by an IgG ELISA and were both determined to be approximately 100 μg/μL. Values represent the average of three independent experiments, and error bars represent SD. (B) Experimental design to assess neutralization efficiency by qRT-PCR. ΔΔCt calculations were performed using a beta-actin TaqMan probe to determine the fold change in capsid RNA expression following ADK8 treatment. This value is designated r_{capsid}. (C) Determination of r_{capsid} values for crude vector preps. Values represent the average of three independent experiments, and error bars represent SEM, calculated as in Livak and Schmittgen.[23] The assay's limit of detection is represented in the dotted line.

consistent with high levels of capsid mosaics, packaging both genome variants at equal frequency. In addition, dilution of the production plasmid stock triggered an increase in the amount of *cap8* detected relatively to *capAnc82* (Figure 3Bii–3Biv). Since AAV8 was shown to produce and purify slightly better than Anc82 (Figure 3Bi; Figures S3 and S4A), such a result was in line with a decrease in capsid mosaicism. In the case of the pSub201 production system, *cap8* represented ~80% of total measured vg in saturation conditions (Figure 3Bii). Taken together, the DSF and qPCR data obtained for the pSub201 libraries suggested that in this production context, higher levels of *cap8* genomes were available for VP production and genome packaging, resulting in capsid mosaics enriched in VP^{AAV8} monomers and *cap8* genomes. Such phenomenon could arise from experimental errors or from an increase in *cap8* replication relative to *capAnc82* in the case where *rep* is provided in *cis*. Together with the results of the capsid/GFP packaging competition assay, the intriguing correlation between capsid mosaic and relative genome titers in our AAV8/Anc82 preps demonstrated a likely increase in packaging fidelity with dilution of input library DNA.

Combined Mosaicism and Cross-Packaging Analysis in Two-Capsid Libraries

We next sought to assess the prevalence of both cross-packaging and mosaicism in AAV8/Anc82 library preps (Figure 1D). To distinguish between the two capsids as sensitively as possible, we took advantage of ADK8, a highly neutralizing monoclonal antibody that binds the variable region VIII (VR-VIII) region of AAV8.[18] AAV8 and Anc82 differ in key residues in this region (Figure 1B), and likely as a result, ADK8 neutralizes AAV8 capsids over 500-fold more efficiently than Anc82 capsids at dilutions of 1:1,250 in an *in vitro* luciferase assay (Figure 4A). With neutralization by ADK8 established as an effective means of differentiating capsids, we devised an assay to determine the genomic contents of neutralized capsids, allowing us to investigate the relationship between cross-packaging and mosaicism. Briefly, ADK8 was used to immunoprecipitate AAV8 and Anc82 particles, and genome abundances were quantified across different fractions by qPCR. Interestingly, whereas ADK8 was found to bind both AAV8 and Anc82 capsids (Figure S7), it only neutralized AAV8 transduction as measured by qRT-PCR. Therefore, we employed a neutralization-based strategy, in which crude preps were treated with ADK8 or isotype control antibody and then used to transduce cells (Figure 4B). RNA was then isolated from transduced cells and assessed for abundance of AAV8 and Anc82 transcripts by qRT-PCR. From this, the fold change in abundance of each transcript in the ADK8-treated cells could be determined and thereby give a metric of the extent to which each particular transcript was neutralized (r_{capsid}).[23] When individually produced AAV8 and Anc82 preps were applied to this assay separately, AAV8 transcript abundance was reduced over 100-fold, whereas Anc82 transcript abundance remained unchanged (Figure 4C). This indicated that the qRT-PCR-based assay maintained the sensitivity of the luciferase-based neutralization assay while distinguishing between differentially packaged vector transcripts, which made it a suitable means to study incorrect packaging of AAV8/Anc82 libraries.

We then applied this assay to analyze libraries produced with decreasing amounts of library plasmid, hypothesizing that this would promote correct packaging within the libraries. A vector library made from two cotransfected capsid plasmids would be likely to contain correctly packaged vectors, along with mosaic capsids and cross-packaged vectors (Figure 5A). Given the different r_{capsid} values of pure AAV8 and Anc82 capsids, we anticipated that different members of this library would be differentially neutralized by ADK8 and therefore help give an indication of the packaging fidelity and mosaicism abundance in the library. In order to approximate the values of r_{capsid} for each possible mosaic capsid, we produced AAV8 and Anc82 vectors in conditions known to generate mosaics and assessed the neutralization of each population by luciferase assay (Figure 5B). Compared to mixtures of the two vectors individually produced and spiked together, vectors produced in mosaic form were far

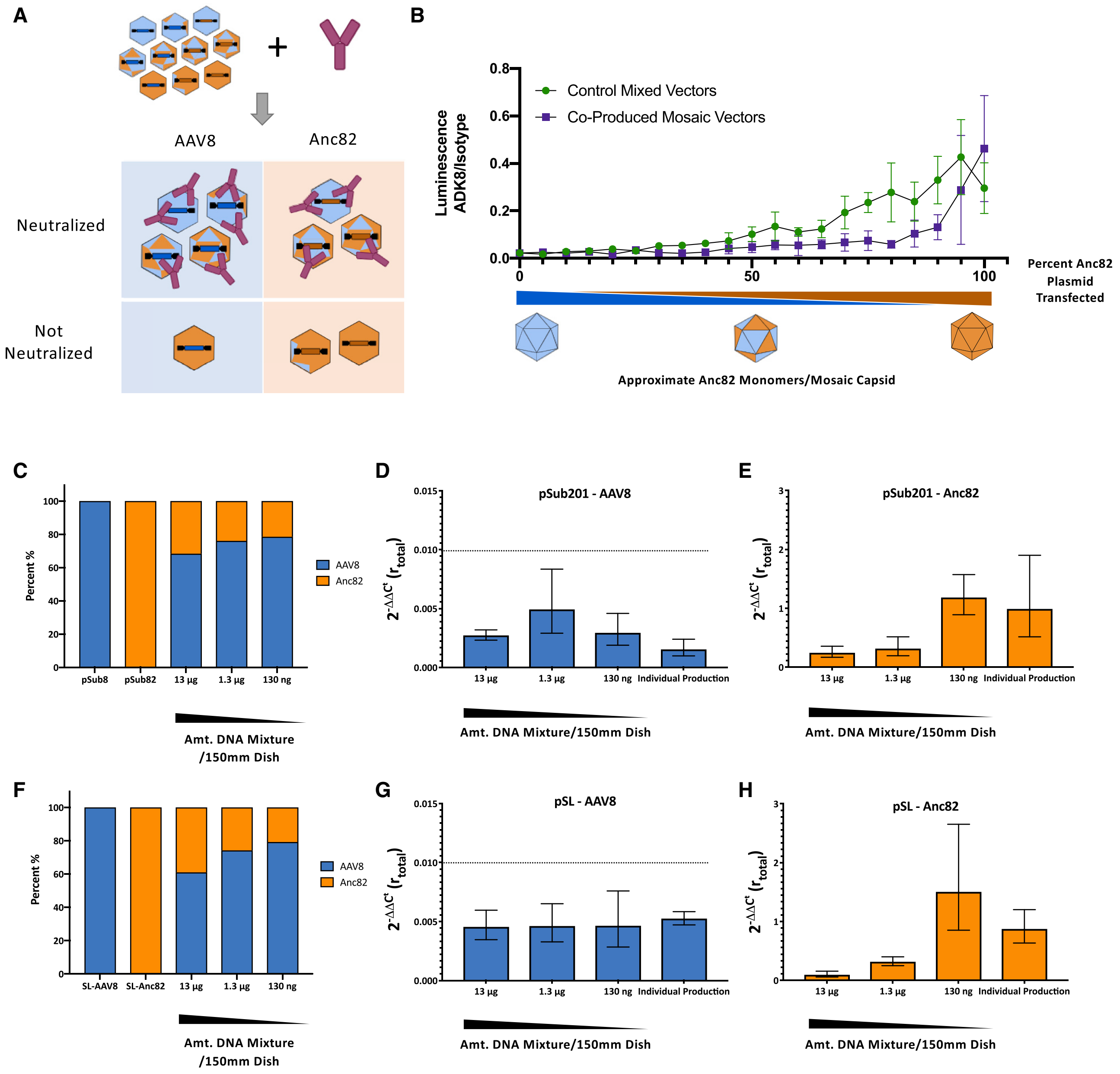

Figure 5. Dilution of Input Capsid DNA Results Improves Packaging Quality in 2-Capsid Libraries

(A) Individual vectors in a mixed population resulting from a mixed transfection are differentially neutralized by ADK8. (B) Neutralization of AAV8/Anc82 capsid mosaics. Varied ratios of AAV8 and Anc82 plasmid were transfected to create mosaic capsids with GFP-luciferase transgenes. Individually produced AAV8 and Anc82 were spiked together at the same ratios to create no-mosaic controls. Neutralization was measured by luciferase assays of transfected HEK293 cells. Error bars represent SD. (C) Relative genome abundance in crude vector preps produced by HEK293 cells transfected with varying amounts of an equimolar pSub201-AAV8/pSub201-Anc82 plasmid mix. (D) $r_{totalAAV8}$ values for populations of vectors produced with decreasing amounts of pSub201-AAV8/pSub201-Anc82 plasmid mixture. ΔΔCt values were derived using AAV8 capsid cDNA and beta-actin abundances in cells transduced with vectors treated with either a negative isotype control antibody or ADK8. A population of vectors mixed together from individually produced AAV8 and Anc82 crude preps was also analyzed as a no-mosaic control. Values represent mean of three independent experiments, and error bars represent SEM.[23] Dashed line represents limit of detection of assay. (E) $r_{totalAnc82}$ values for populations tested in (D). All computations are identical to those used to determine $r_{totalAAV8}$, only Anc82 capsid cDNA abundance was measured. (F) Relative genome abundance in crude vector preps produced by HEK293 cells transfected with varying amounts of an equimolar pSL-AAV8/pSL-Anc82 plasmid mix. Methods and computation are identical to those employed in (C). (G) $r_{totalAAV8}$ values for populations of vectors produced with decreasing amounts of pSL-AAV8/pSL-Anc82 plasmid mixture. Methods and computation are identical to those employed in (D). (H) $r_{totalAnc82}$ values for populations tested in (G). All computations are identical to those used to determine $r_{totalAAV8}$, only Anc82 capsid cDNA abundance was measured.

more readily neutralized by ADK8, indicating neutralization of mosaics, relative overabundance of AAV8 capsids, or some combination thereof. In any case, the r_{total} values for each population, as determined by qRT-PCR, could be used to estimate the overall packaging fidelity of the library (Figure S8).

Three dilutions of an equimolar pSub201-AAV8/pSub201-Anc82 plasmid mix were used to generate three AAV8/Anc82 libraries (Figure 5C). Abundance of each genome was in line with prior results (Figure 3B). Equivalent titers of these libraries were then treated with either ADK8 or an isotype control, and neutralization of AAV8 and Anc82 genome transcripts was assessed by qRT-PCR to obtain a r_{total} value for each genome population. We found that the expression of AAV8 transcripts from ADK8-treated samples was undetectable across libraries and indistinguishable from a control library composed of individually produced AAV8 and Anc82 preps (Figure 5D). However, the r_{total} value for Anc82 genomes in these populations was diminished significantly in the library generated with the most input library plasmid but increased to be indistinguishable from the control library as the amount of input plasmid decreased (Figure 5E). This indicated that a significant portion of Anc82 genome-containing capsids were neutralized by ADK8, suggesting that they were mispackaged into capsids containing significant amounts of AAV8 monomers. These experiments were repeated with libraries produced in the pSL format with supplemental *rep* (Figure 5F). The r_{total} values for AAV8 genomes were once again below the limit of detection (Figure 5G). Anc82 r_{total} values showed similar trends to those in the pSub201 libraries, although it appeared that mispackaging was even more prevalent in libraries produced with more input plasmid (Figure 5H). Overall, these results appear to indicate that fully cross-packaged particles are exceptionally rare, as cross-packaged AAV8 genomes were not detectable, and that decreases in capsid mosaicism are responsible for the increased r_{total} values that accompanied dilution of Anc82 genomes. These findings are consistent with the results of the DSF assay, both confirming a lower rate of mosaic formation in WT plasmid libraries and demonstrating that 100-fold dilution of input library DNA leads to limited mosaic formation in the resulting capsid library.

Capsid Mosaic Homogeneity: rAAV versus WTAAV

Recently, Nonnenmacher et al.[14] provided compelling evidence that in saturation conditions, cross-packaging and mosaicism were favored in a recombinant production context but limited in a WT production context. Our results did not align with these conclusions, as we could detect cross-packaging and mosaic formation in simple AAV libraries produced at high *rep/cap* levels (Figures 2 and 3). Nonetheless, both studies relied on very different characterization methods. In particular, Nonnenmacher et al.[14] investigated the process of capsid formation by the means of infectivity, western blot, and binding assays, whereas DSF was used in our study. In order to determine whether these discrepancies could be related to differences in sensitivity between both capsid mosaic detection approaches, we produced a recombinant AAV8 (rAAV8)/Anc82 prep (CMV.EGFP.t2a.luciferase.simian virus 40 [SV40]) in saturation conditions and purified it as previously described. Following quality control (Figures S3–S5), the DSF profile of rAAV8/Anc82 was generated and compared to that obtained for the pSL and pSub201 AAV libraries (Figure 6A). In line with the pSL and pSub201 libraries produced at 13 μg *rep/cap* per dish (Figures 3Ai and 3Aii), rAAV8/Anc82 yielded four distinct transitions (Figure 6A). However, the amplitudes of the two intermediate peaks, centered on 78°C and 85°C, were much higher than those of the low (73°C) and high (90°C) temperature peaks, suggesting higher levels of VP^{AAV8}:VP^{Anc82} interfaces than VP^{AAV8}:VP^{AAV8} and VP^{Anc82}:VP^{Anc82} interfaces in recombinant mosaics. This result possibly indicated that in contrast with the broad spectrum of VP^{AAV8}:VP^{Anc82} stoichiometries obtained for WTAAV preps (Figures 5 and 6B), rAAV mosaics appeared to be relatively homogeneous in terms of VP composition (Figure 6B). Of note, the right peak obtained for the rAAV prep was centered on a temperature that was 1.4°C lower than that obtained for WTAAVs. It is not excluded that high levels of VP^{AAV8}:VP^{Anc82} could destabilize residual VP^{Anc82}:VP^{Anc82} interfaces within capsid mosaics produced in a recombinant context.

To provide further evidence that these differences in DSF fingerprints reflected variations in capsid VP composition homogeneity (Figure 6B), we showed that the thermal stability profile of pSub201-AAV8/Anc82 could be recapitulated by subjecting mixes of pSub201-AAV8, pSub201-Anc82, and pAAV-AAV8/Anc82 particles to DSF. Results of this experiment are detailed in the Supplemental Information (Figure S9).

Lastly, we hypothesized that the differences in VP composition observed between WT and rAAV8/Anc82 particles resulted from ITR-dependent variations in VP expression dynamics. To test this hypothesis, we fused the *mCherry* and *EGFP* genes to *cap8* and *capAnc82*, respectively, in both the pSub201 and pAAV backbones (Figure 6C). *cap* and fluorescent protein-encoding genes were separated by a T2A peptide sequence, allowing for the expression of VPs and fluorescent proteins at a ratio 1:1 from the same p40 transcripts (Figure 6C).

The fluorescence profiles obtained upon transfection with either AAV8.T2A.mCherry or Anc82.T2A.EGFP were independent of the presence or absence of ITRs (Figure 6D; AAV8 and Anc82 conditions). Upon cotransfection of HEK293 cells with AAV8.T2A.mCherry and Anc82.T2A.eGFP, in the absence of ΔF6, nearly identical fluorescence profiles were obtained from pSub201 and pAAV (Figure 6D; AAV8 + Anc82 [No Ad] condition). These profiles were characterized by a majority of nonfluorescent cells (~75%), accompanied by ~15% of cells exhibiting similar EGFP and mCherry intensity levels, resulting in linear scatterplots (Figure 6D; No Ad condition). Since HEK293 cells constitutively express the Ad5 *E1* gene,[24] such a result was consistent with basal coexpression of VP^{AAV8} and VP^{Anc82} following p4 transactivation and subsequent expression of Rep2. Interestingly, in the presence adenovirus helper plasmid (Figure 6D; No Ad condition), two different fluorescence profiles could be observed, depending on the presence or absence of ITRs. Both production systems

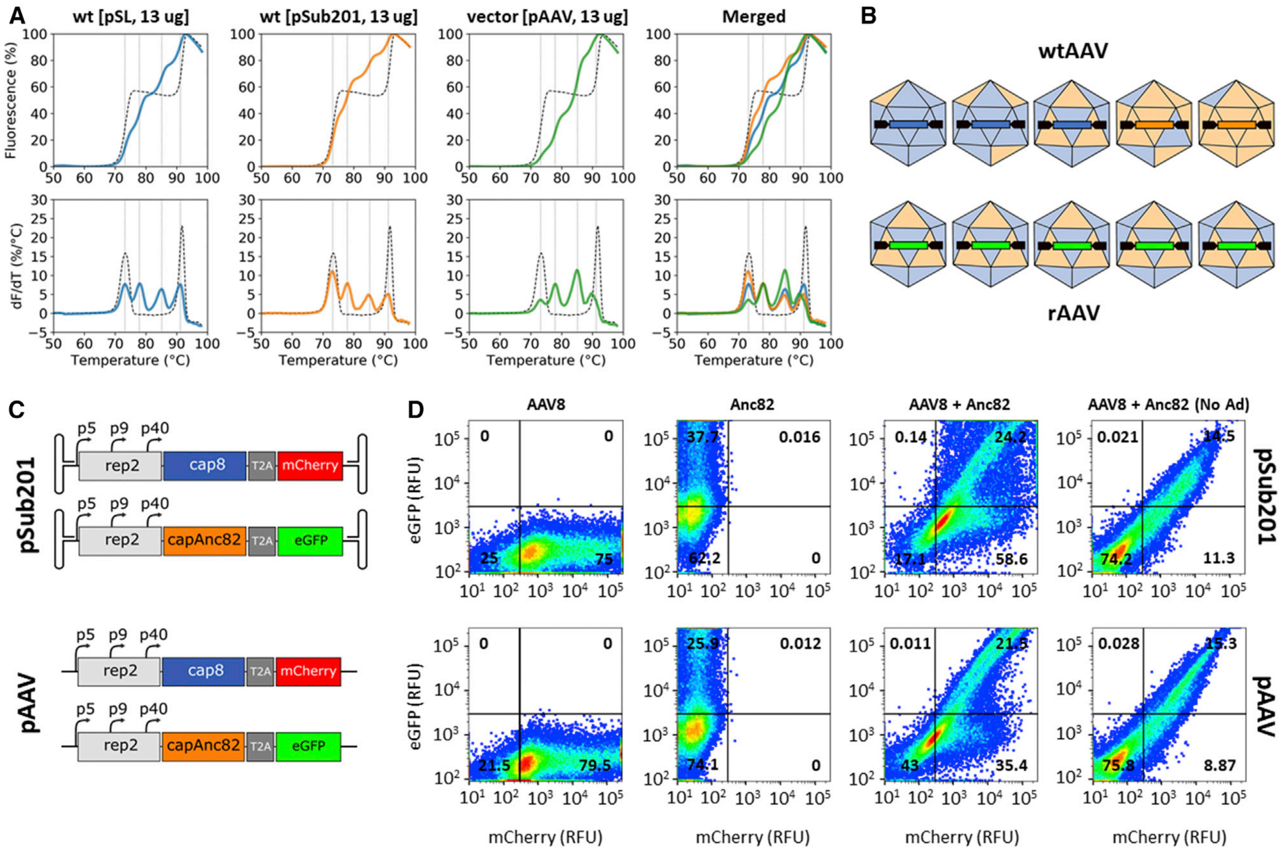

Figure 6. Capsid Mosaic Homogeneity in a WT and Recombinant Production Context

(A) Thermal stability profiles of AAV preps produced in saturation conditions in a WT (pSL and pSub201 system) and a recombinant (pAAV) context. (B) Cartoon representation of WTAAV and rAAV capsid mosaics, illustrating differences in VP^{AAV8}:VP^{Anc82} stoichiometry and genome content. (C) AAV-Fluo production systems, obtained through insertion of T2A peptide sequences and fluorescent protein-encoding genes into pSub201 and pAAV plasmids. (D) FACS analysis of EGFP and mCherry expression in HEK293 cells, transfected with AAV-Fluo plasmids (13 μg/plate in total), in the presence (pSub201) or absence (pAAV) of ITRs. Cells were analyzed 48 h post-transfection.

yielded 21%–25% EGFP/mCherry double-positive cells (Figure 6D; No Ad condition). In the absence of ITRs (pAAV system), most of the double-positive cells exhibited similar EGFP and mCherry intensity levels, resulting in a relatively linear scatterplot. In the presence of ITRs (pSub201 system), a broader range of EGFP/mCherry intensity ratios could be observed within the cell population expressing both fluorescent proteins. Such a result suggested that the presence of ITRs could favor the overexpression of either VP^{AAV8} or VP^{Anc82} capsid components in cotransfected cells, ultimately leading to preps of capsid mosaics with heterogeneous VP8:VPAnc82 stoichiometries and high genome-VP correlation (Figure 6B).

Effects of Packaging Quality on *In Vivo* Library Screens

Finally, we assessed the quality of *in vivo* barcoded AAV screening results from libraries produced in the same high, medium, and low input capsid DNA conditions as compared to an individually produced, pooled library (Figure 7A). In comparison to the starting plasmid pool, all three libraries had slightly varied abundances of each member, but each was roughly consistent with the individually produced and pooled vectors (Figure 7B). All four libraries were injected retro-orbitally into mice (n = 5 per condition) at a dose of 1E+11 total genome copies, and livers were harvested 7 days postinjection for DNA barcode extraction and sequencing using the Illumina MiSeq platform. All groups showed similar vector genome abundance in livers (Figure S10). The individually produced control library showed an increase in barcodes corresponding to AAV8 and AAV1 capsids (Figure 7C). The fold enrichment of each liver barcode over the input vector was calculated, and the overall distribution of these values most closely approximated the individual control library in the low-input DNA library (Figure 7D). This visual observation was confirmed with computation of Euclidian distance between each distribution (Figure 7E). Taken together with our previous results, these data indicate that AAV libraries produced with lower amounts of input capsid library yield higher-quality data when used in screens, likely as a result of enhanced packaging fidelity and limited mosaicism in these libraries.

DISCUSSION

The use of pooled, multiplex libraries of AAV capsid variants is a common approach used to identify AAV capsids with novel

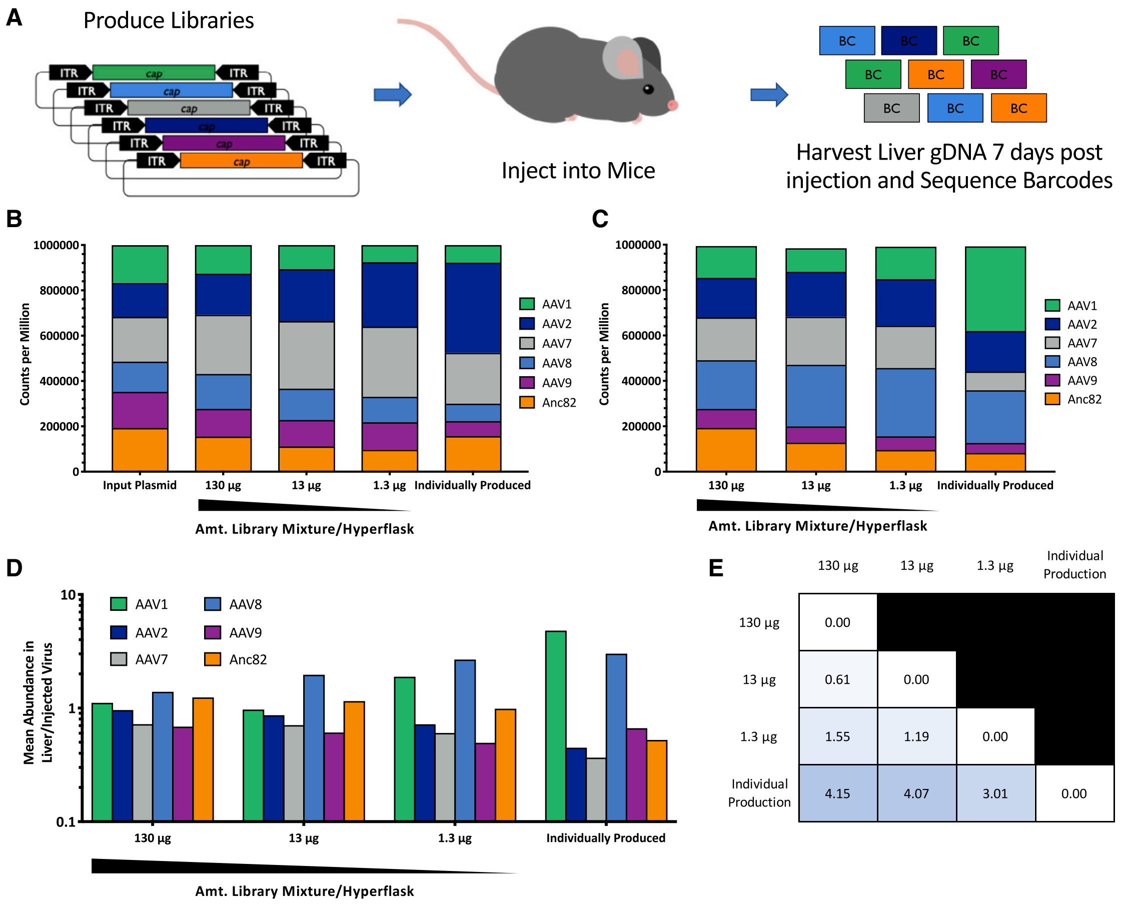

Figure 7. Dilution of Input Capsid DNA Results in Higher Quality Multiplexed Libraries

(A) Experimental design. AAV1, AAV2, AAV7, AAV8, AAV9, and Anc82 capsid sequences tagged with uniquely identifying barcodes in pSL vectors were assembled into an equimolar library and used to generate a high titer library prep. In parallel, plasmids were used to generate individual preps that were then combined into a control library. All libraries were injected into mice, and livers were harvested 7 days following injection. Barcodes were isolated from liver genomic DNA and sequenced. (B) Distribution of barcode abundances in counts per million in input plasmid library (left) multiplexed vector libraries, generated using the plasmid library (middle) and a control library of individually produced and spiked vectors (right). (C) Average of barcode abundances in liver gDNA in counts per million in mice injected with each multiplexed vector library (left) and individually produced and spiked vector library (right). n = 5 mice were injected with each library. (D) Average fold change in abundance of each barcode in liver gDNA over injected vector library. (E) Euclidian distance between the distribution of fold changes for each library.

properties, such as in directed evolution. These methods rely on the determination of the identity of vg encoding the capsid sequence and/or another identifier as a proxy of the protein capsid that confers the phenotype selected. Here, we aimed to quantify to which extent this linkage between vg and the protein capsid is maintained. Lack of this linkage introduces a noise function into any screen, and its level will determine whether a screen can be performed reproducibly and therefore, will be predictive.

Previously, both cross-packaging and mosaicism have been described as mechanisms that dissociate this genotype-phenotype linkage in pooled, multiplex libraries. Whereas cross-packaging is the full mismatch of the genome and capsid, a capsid mosaic describes the state of multiple, different VP monomers being integrated into a virion. The properties of such mosaic virion are not predictable and in specific pairings, have been shown to lead to incompatible, intermediate, and synergistic phenotypes.[17] Whereas initial observations on these processes were made in the context of vector production (without the capsid being encoded on the vg), previously, Nonnenmacher et al.[14] convincingly reported that cross-packaging and possibly, by extension, mosaic formation are limited in the context of a WTAAV production due to a mechanism of preferential packaging of "self" genomes.

Here, we studied the quantitative nature of both mosaicism and cross-packaging in the context of HEK293 cotransfection AAV production, using libraries of increasing complexity and relevance. Our aims were to quantify the level of both of these processes and identify approaches to control them that could be employed in production of larger libraries intended for functional screens. Results from our

cross-packaging competition assay demonstrate that at high plasmid levels, AAV capsids do not preferentially package their genome (Figure 2). In addition, our DSF data also indicate the presence of capsid mosaics in the AAV libraries produced in saturation conditions (Figure 3). At first sight, these conclusions do not align with those formulated by Nonnenmacher and colleagues,[14] who reported low cross-packaging and mosaic levels in AAV libraries. Nevertheless, this apparent contradiction can be nuanced by the results of our qRT-PCR/ADK8 neutralization assay (Figure 5), suggesting the following: that (1) the capsid mosaics formed in AAV libraries are highly heterogeneous in terms of VP^{AAV8}:VP^{Anc82} stoichiometry and (2) mosaics packaging a given *cap* gene have a high probability of being enriched in the VPs that this gene encodes. Such interpretations are in line with the results of the binding and infectivity experiments presented by our colleagues.[14] This high VP-genome correlation within mosaic populations would also explain how novel, tissue-specific AAV variants could be discovered via directed evolution of AAV libraries produced in saturation conditions.[8,25]

We also provide evidence that the presence or absence of ITRs influences the dynamics of VP production within transfected cells, resulting in mosaic populations with various VP stoichiometry profiles (Figure 6). Our fluorescence-activated cell sorting (FACS) data suggest that in a WT production context, most VP-producing cells express both VP^{AAV8} and VP^{Anc82}, muddling the hypothesis of a "pioneer" vg overtaking the cell, following its initial expression from an infectious clone plasmid.[14] Nonetheless, it remains tempting to speculate that genome replication is a key determinant of the differences in capsid mosaic composition observed between the WT and recombinant production contexts. It is not unlikely that transfected cells internalize both *rep/cap* variants at slightly different ratios. In this scenario, replication of ITR-containing vgs would trigger the exponential amplification of the initial delta between *cap8* and *capAnc82* sequence levels. This would result in a broad spectrum of *cap8:capAnc82* stoichiometries and VP expression profiles within producing cells, ultimately leading to preps of capsid mosaics with various VP^{AAV8}:VP^{Anc82} stoichiometries and a high VP-genome correlation (Figure 6B). It remains to be seen how this phenomenon would manifest in cells transfected with more complex and relevant libraries.

In addition, it is interesting to challenge our AAV8/Anc82 experimental model, not only in terms of simplicity but also in the biology of the two capsids used. AAV capsids were shown to assemble in the nucleus in a serotype-dependent fashion.[26,27] As a result, the extent to which VPs from two distinct serotypes can oligomerize within producing cells may be highly dependent on serotype identity. Assembled AAV8 capsids were shown to accumulate in both the nucleoplasm and the nucleolus.[27] Anc82 was recently shown to be strongly dependent on the AAV receptor (AAVR) for transduction[28] and assembly-activating protein (AAP) for capsid formation,[29] yet its assembly profile remains unknown and should be investigated. Both serotypes may also exhibit different genome replication and packaging efficiencies. Therefore, cross-packaging and mosaic levels in AAV8/Anc82 library preps may not accurately reflect the behavior of more complex AAV libraries, containing thousands of variants with higher VP sequence homology (>99.5%) and lower functional diversity in terms of capsid assembly, genome replication, and DNA packaging.

Finally, this work has direct implications the generation and screening of AAV capsid libraries. Dilution of the input plasmid library in transfections has been widely used to promote high-quality packaging using the rationale that dilution reduces the number of plasmids that are ultimately taken up by a single cell. Initial dilution experiments in Figure 2 demonstrate that ITR-flanked capsid plasmids are capable of producing vector even when diluted over 100-fold relative to other transfection components, which is in surprising contrast to ITR-free plasmids (Figure S2). As the experiments described in Figure 7 indicate, dilutions of plasmid libraries up to 100-fold less than typically used in AAV production can be used to generate high-quality viral libraries, with degree of dilution corresponding to overall quality. We suggest that researchers seeking to produce optimal libraries dilute their plasmid libraries 10- to 100-fold when producing AAV libraries, taking into consideration the screening applications and overall assembly and production abilities of the library. If a library produces poorly, more modest dilutions may be necessary to preserve vector yield. Of additional importance, we note that known production characteristics of individual AAV capsids are not necessarily conserved when produced in a pooled format. Figure 7B indicates that dilution increases the abundance of AAV2 in a pooled population, while decreasing AAV8, whereas the field has long been aware that AAV8 is a superior producer to AAV2 when the two capsids are produced separately.[30] We have also seen similar results with these two capsids in a variety of much larger library productions (data not shown). For this reason, we do not recommend pooled library screens for production phenotypes, although the phenomenon, in itself, is an interesting avenue for further study.

Whereas the mechanisms governing multiplexed AAV library production remain elusive, we have demonstrated that the prevalence of cross-packaging and mosaicism is highly dependent on the conformation and abundance of input plasmid library. With a collection of novel and sensitive assays, we have shown that even relatively modest dilutions of input plasmid greatly enhance VP homogeneity and packaging fidelity. Practically speaking, we have confirmed ideal methods for production of the multiplexed AAV library that maximize titers while minimizing noise, therefore improving the impact of future screens.

MATERIALS AND METHODS

AAV Production Plasmid Generation

pSub201-r2cAnc82 was generated by PCR cloning. The 5′-KpnI-r2cAnc82-AgeI-3′ insert was PCR amplified from pAAV2/Anc82, using the primers listed in Table S2, and ligated to the pSub201-r2c8 vector following digestion with KpnI/AgeI. pSub201-r2c8-t2a-mCherry and pSub201-r2cAnc82-t2a-EGFP were generated by restriction/ligation. Both EagI-cap8-t2a-mCherry-AgeI and

SbfI-*capAnc82*-t2a-EGFP-AgeI were synthesized (Genewiz, South Plainfield, NJ, USA) and ligated to pSub201-r2c8 and pSub201-r2cAnc82 vectors, respectively, following digestion with EagI/AgeI or SbfI/AgeI. To facilitate the cloning, one AgeI restriction site was removed from the *cap8* sequence of pSub201-r2c8. pAAV-r2cAnc82-t2a-EGFP was cloned by restriction/ligation. The HindIII-r2cAnc82-t2a-EGFP-SpeI insert was obtained by digestion of pSub201-r2cAnc82-t2a-EGFP with HindIII/SpeI and ligated to the pAAV-r2cAnc82 vector, following digestion with the same enzymes. pAAV-r2c8-t2a-mCherry was generated by PCR cloning. The SpeI-r2c8-t2a-mCherry-HindIII was PCR-amplified from pSub201-r2c8-t2a-mCherry, using the primers listed below. The SpeI-r2c8-t2a-mCherry-HindIII insert and pAAV-r2cAnc82 vector were digested with SpeI/HindIII and further ligated. All ligation reactions were run for 15 min, at room temperature, using the Anza T4 DNA Ligase Master Mix (IVGN2104, Thermo Fisher Scientific). Restriction enzymes were purchased from New England Biolabs (NEB; Ipswich, MA, USA) and Thermo Fisher Scientific.

ITR-free plasmids were amplified at 37°C in XL1-blue electroporation competent (#200228; Agilent Technologies, Santa Clara, CA, USA) or DH5α chemically competent (18258012; Thermo Fisher Scientific, Waltham, MA, USA). ITR-containing plasmids were amplified at 30°C in Stbl4 electroporation competent (11635018; Thermo Fisher Scientific) or NEB stable competent *Escherichia coli* (C3040I; NEB). Following plasmid extraction, ITR integrity was verified by restriction analysis using SmaI + EcoNI (1% agarose gel electrophoresis), MscI + EcoNI (1% agarose gel electrophoresis), PvuII + XbaI (3% agarose gel electrophoresis), and PvuII + AgeI (3% agarose gel electrophoresis).

Cell Culture

HEK293 cells (ATCC) were maintained in DMEM (Corning), supplemented with 10% fetal bovine serum (GE Healthcare) and 100 IU/ml penicillin/streptomycin (Corning). Cells were grown in a humidified incubator at 37°C with 5% CO_2.

AAV Library Production and Purification

For AAV library preps, AAVs were produced by PEI transfection of 70%–95% confluent-adherent HEK293 cells. The plasmid mixes used for the different AAV preps are detailed in Table S3.

For one, 15-cm dish, plasmid mixes were prepared in 1 mL serum-free DMEM (4.5 g/L glucose, 1% penicillin/streptomycin), vortexed for 10 s. 1 mL PEI Max (Polysciences, Warrington, PA, USA) or PEIpro (Polyplus transfection, Illkirch, France) solutions were prepared in serum-free DMEM and vortexed for 10 s. PEI and plasmid solutions were mixed (2 mL total), vortexed for 20 s, and incubated at room temperature for 15 min (final DNA:PEI = 1:1.375). Following incubation, each transfection mix was added to 18 mL serum-free DMEM (4.5 g/L glucose, 1% penicillin/streptomycin). Cell medium was aspirated and replaced with the final 20-mL transfection mix. Cells were further incubated for 72 h at 37°C, 5% CO_2. This protocol was scaled up (520 μg total DNA per 10-layer hyperflask [Corning]) or down (6 μg total DNA per well of a 6-well plate), depending on the production needs. To generate small-scale crude AAV preps, cells and supernatant were harvested from 6-well plates at 72 h post-transfection, subjected to three freeze/thaw cycles, followed by centrifugation at 14,000 *g* for 10 min and supernatant collection.

AAV Production

Vectors were produced in one of three possible ways. For small-scale productions, approximately 6 μg of total DNA in 100 μL of serum-free DMEM was mixed with 100 μL of DMEM containing polyethylenimine (PEI) Max (Polysciences) at a 1.375:1 PEI:DNA (w/w) ratio and allowed to incubate at room temperature for 15 min. Each mixture was then added into a well of a 6-well plate containing 90% confluent HEK293 cells in 2 mL serum-free DMEM. Cells and supernatant were harvested after 72 h and subjected to three freeze/thaw cycles, followed by centrifugation at 14,000 *g* for 10 min to generate crude vector preps. Mid-scale productions followed a similar scaled-up protocol in which either 47 μg of total DNA was transfected into a 15-cm dish (for cross-packaging analysis) or 52 μg of total DNA was transfected into a 15-cm dish (for DSF and mosaicism analysis) with PEI Max or PEIpro (Polyplus transfection, Illkirch, France), respectively. Preps used for cross-packaging analysis used slightly less total DNA to conserve molar amounts of *cap* elements across different production schemes (WT pAAV, pSL, and pSub201). See Tables S3 and S4 for descriptions of respective transfection mixes. Large-scale AAV8/Anc82 library preps (10-layer hyperflasks or ten to fifteen, 15-cm dishes) were purified by tangential-flow filtration, combined to iodixanol gradient ultracentrifugation, or by affinity chromatography using the POROS-CaptureSelect AAVX Affinity Resin (A36739; Thermo Fisher Scientific). Large-scale productions used for animal experiments were carried out by the Grousbeck Gene Therapy Center Gene Transfer Vector Core and involved transfection of up to 520 μg of total DNA into a 10-layer hyperflask, followed by purification of the lysate using tangential-flow filtration and iodixanol gradient ultracentrifugation.

qPCR

vg levels were quantified by qPCR, using primers and probes targeting ITR, *cap8*, *capAnc82*, or transgene sequences. 3 μL of AAV prep was incubated for 45 min, at 37°C, with 20 U of DNaseI (04716728001; Roche, Basel, Switzerland). Digested samples were then supplemented with 20 μL of 20 mg/mL proteinase K (740506; MACHEREY-NAGEL) and incubated at 70°C for 20 min. vgs were further extracted using the NucleoSpin RNA Virus kit (MACHEREY-NAGEL). qPCR was performed with a StepOnePlus Real-Time PCR (Life Technologies, Carlsbad, CA, USA). qPCR reactions were run in a final volume of 20 μL, containing the primers/probe PCR Master Mix (TaKaRa, Kusatsu, Japan) and 5 μL of template DNA. For ITR titration, the standard plasmid was prepared in accordance with D'Costa et al.[21]

DSF

DSF assays were all run at constant vp per well, as indicated in the figure legends. 500 μL SYPRO Orange, 50 times, was prepared using

PBS2+ (21-030-CV; Corning) as a solvent. 96-Well plates were loaded with 45 μL sample, supplemented with 5 μL SYPRO Orange, 50 times. PBS2+ and 0.25 mg/mL lysozyme (L6876; Sigma-Aldrich) solutions were used as negative and positive controls, respectively. Plates were sealed, centrifuged at 3,000 rpm for 2 min, and subsequently loaded into a StepOnePlus Real-Time qPCR instrument (Life Technologies). Samples were incubated at 25°C for 2 min prior to undergoing a temperature gradient (25°C–99°C, ∼9°C/10 min, "continuous" mode with gradient set to 1%) while monitoring the fluorescence of the SYPRO Orange dye using the ROX filter cube of the qPCR instrument. To improve the signal-to-noise ratio, fluorescence signals were all smoothed by convolution, with a moving average box of 5, using an in-house Python script. Smoothed fluorescence signals F were normalized between 0% and 100%, and Tms were defined as the temperature for which the numerical derivative, change in fluorescence level/change in temperature (dF/dT), reached its maximum.

Luciferase Neutralization Assay

One day prior to neutralization, HEK293 cells were plated at 20,000 cells/well into black 96-well plates previously coated with 0.01% poly-L-lysine solution (A-005-C; Sigma). The next day, vector and antibody were added to DMEM to concentrations of 2.0E+9 genome copies (GC)/mL and approximately 80 μg/mL (1:1,250 dilution), respectively, and allowed to incubate at 37°C for 1 h. Antibodies used were either a mouse immunoglobulin G (IgG)2A kappa (14-4724-81; Thermo Fisher Scientific) or ADK8 (03-652160; American Research Products). Following incubation, media on cells were replaced with 50 μL of the mixture in each well and allowed to incubate for an additional hour, at which point 150 μL of serum-containing media was added. After 48 h, media were removed, and cells were lysed with 20 μL/well, one time Reporter Lysis Buffer (E1941; Promega) and frozen. Once thawed, firefly luciferase expression was measured using a Synergy H1 Hybrid Multi-Mode microplate reader and 100 μL/well luciferin buffer (200 mM Tris [pH 8], 10 mM $MgCl_2$, 300 μM ATP [A2383-5G; Sigma]; one time Pierce luciferase signal enhancer [16180; Thermo Fisher Scientific]; and 150 μg/mL D-luciferin [L2916; Thermo Fisher Scientific]).

qRT-PCR Neutralization Assay

One day prior to neutralization, HEK293 cells were plated at a density of 120,000 cells/well in 24-well plates. Vector and antibody mixtures were prepared and incubated as indicated above, and then 300 μL of the mixture was added per well. Cells were allowed to incubate for 1 h, after which 900 μL of serum-containing media was added per well. Following 48 h of incubation, media were removed, and cells were harvested in 500 μL TRIzol (15596026; Thermo Fisher Scientific). To extract RNA, 100 μL of chloroform was added to each sample, and samples were vortexed and centrifuged at 12,000 *g* for 15 min at 4°C. Aqueous phases were extracted and added to 250 μL isopropanol, supplemented with 1 μL GlycoBlue coprecipitant (AM9516; Thermo Fisher Scientific), vortexed, and allowed to incubate for 10 min before centrifugation at 12,000 *g* for 10 min at 4°C. The pellet was washed with 1 mL of 70% ethanol, dried, and resuspended in 50 μL of nuclease-free water. Samples were then treated using the DNA-free DNA removal kit (AM1906; Thermo Fisher Scientific). 1 μg of DNase-treated RNA was used to generate cDNA with the SuperScript IV First-Strand Synthesis System (18091200; Thermo Fisher Scientific), priming with random hexamers. TaqMan qRT-PCR was used to quantify B-actin, AAV8, and Anc82 transcript copies in each sample; 62.5 ng of cDNA was used per 25 μL reaction volume. Oligonucleotides used can be found in Table S5. Primer efficiencies can be found in Figure S11.

Flow Cytometry

In order to investigate the dynamics of VP coexpression in the absence or presence of ITRs, HEK293 cells were transfected as described above, using the following plasmid mixes: for pSub201 transfections, cells were transfected with 26 μg ΔF6 Helper, 6.5 μg AAV8-T2A-mCherry, 6.5 μg Anc82-T2A-EGFP, and 13 μg pSEAP2, whereas for pAAV transfections, cells were transfected with 26 μg ΔF6 Helper, 6.5 μg AAV8-T2A-mCherry, 6.5 μg Anc82-T2A-EGFP, and 13 μg pCMV-LacZ. For both the pSub201 and pAAV systems, three control conditions were added (13 μg AAV8-T2A-mCherry only, 13 μg Anc82-T2A-EGFP only, and both plasmids [6.5 μg each] in the absence of ΔF6 helper). Cells were incubated for 48 h at 37°C, 5% CO_2, and subsequently prepared for flow cytometry analysis. HEK293 cells were trypsinized, transferred into a 15-mL Falcon tube, rinsed with 5 mL ice-cold PBS, and incubated for 7 min in 1% ice-cold paraformaldehyde (PFA). Following three rinses in 5 mL ice-cold PBS, cells were resuspended in 950 μL PBS and transferred to FACS tubes. Fluorescent levels were analyzed using a BD FACSAria III cell sorter (BD Biosciences, Franklin Lakes, NJ, USA). At least 100,000 events were collected for each sample. Flow cytometry data were analyzed using the FlowJo software package.

Animal Studies

All animal studies were performed in accordance with protocols approved by the Institutional Animal Care and Use Committee (IACUC) at Schepens Eye Research Institute. 6- to 8-week-old C57BL/6 male mice were procured from The Jackson Laboratory. After being anesthetized with isoflurane, each animal was injected retro-orbitally with a total of 1.0E11 vector genome copies in 100 μL. Mice were sacrificed 7 days after injection, and livers were harvested and flash frozen. Frozen caudate lobes were pulverized for 45 s using the SPEX SamplePrep 2010 Geno/Grinder set to 1,750 rpm and resuspended in 1 mL of Buffer RLT (79216; QIAGEN), supplemented 1:100 with 2-mercaptoethanol (M3148-100; Sigma). 50 μL of lysate was used as input for the QIAGEN AllPrep DNA/RNA Mini Kit (80204; QIAGEN). 50 ng of total genomic DNA (gDNA) was used as input for a barcode amplification PCR using KAPA HiFi Hotstart Readymix (NC0295239; Thermo Fisher Scientific). Amplified barcodes were extracted from a 1.5% agarose gel using the QIAquick Gel Extraction Kit (28704; QIAGEN), and 2.5 μL of each sample was used as input in a secondary indexing PCR reaction using the Nextera XT Index Kit v2 (FC-131-2001; Illumina) and KAPA Readymix. Reactions

were cleaned using AMPure XP beads (A63881; Beckman Coulter), pooled, and sequenced using a MiSeq Reagent Micro Kit v2 (MS-103-1002; Illumina), according to the manufacturer's protocols.

SUPPLEMENTAL INFORMATION

Supplemental Information can be found online at https://doi.org/10.1016/j.omtm.2019.11.014.

AUTHOR CONTRIBUTIONS

Conceptualization, L.H.V., E.A., S.P., and P.F.S.; Methodology, Validation, and Formal Analysis, P.F.S. and S.P.; Computational Support, E.Z.; Resources, E.T., F.N., F.B., E.A.-M., R.X., M.P.-B., M.B., and N.J.; Writing – Original Draft, P.F.S. and S.P.; Writing – Review & Editing, L.H.V., E.A., S.P., and P.F.S.; Supervision, L.H.V., E.A., M.P.-B., and E.A.-M.; Funding Acquisition, L.H.V., E.A., and O.A.

CONFLICTS OF INTEREST

L.H.V. is an inventor on several patents related to AAV gene therapy, including AncAAV variants, AAV9, and method patents, which are licensed to several biopharma companies. L.H.V. further receives funding from Lonza/Houston, Selecta Biosciences, and Solid Biosciences, licensors to AncAAV technology. L.H.V. is a consultant to Nightstar, Selecta, Akouos, and Exonics and a founder of Akouos. L.H.V. has a financial interest in TDTx, a company developing AAV gene therapies; he is an inventor of technology related to AAV gene therapy, a founder of the company, and also serves on its Board of Directors. L.H.V.'s interests were reviewed and are managed by MEE and Partners HealthCare in accordance with their conflicts of interest policies.

ACKNOWLEDGMENTS

We thank Carmen Unzu, Jennifer Santos, and Reynette Estelien for support in the *in vivo* studies and the staff of the Gene Transfer Vector Core for vector production. We also thank the Cytocell Flow Cytometry Core Facility (Nantes University). This work was funded by Lonza Houston and Giving\Grousbeck (to L.H.V.).

REFERENCES

1. Rose, J.A., Maizel, J.V., Jr., Inman, J.K., and Shatkin, A.J. (1971). Structural proteins of adenovirus-associated viruses. J. Virol. *8*, 766–770.
2. Berns, K.I. (1990). Parvovirus replication. Microbiol. Rev. *54*, 316–329.
3. Podsakoff, G., Wong, K.K., Jr., and Chatterjee, S. (1994). Efficient gene transfer into nondividing cells by adeno-associated virus-based vectors. J. Virol. *68*, 5656–5666.
4. Naso, M.F., Tomkowicz, B., Perry, W.L., 3rd, and Strohl, W.R. (2017). Adeno-Associated Virus (AAV) as a Vector for Gene Therapy. BioDrugs *31*, 317–334.
5. Fitzpatrick, Z., Leborgne, C., Barbon, E., Masat, E., Ronzitti, G., van Wittenberghe, L., Vignaud, A., Collaud, F., Charles, S., Simon Sola, M., et al. (2018). Influence of Pre-existing Anti-capsid Neutralizing and Binding Antibodies on AAV Vector Transduction. Mol. Ther. Methods Clin. Dev. *9*, 119–129.
6. Zincarelli, C., Soltys, S., Rengo, G., and Rabinowitz, J.E. (2008). Analysis of AAV serotypes 1-9 mediated gene expression and tropism in mice after systemic injection. Mol. Ther. *16*, 1073–1080.
7. Ayuso, E. (2016). Manufacturing of recombinant adeno-associated viral vectors: new technologies are welcome. Mol. Ther. Methods Clin. Dev. *3*, 15049.
8. Grimm, D., Lee, J.S., Wang, L., Desai, T., Akache, B., Storm, T.A., and Kay, M.A. (2008). In vitro and in vivo gene therapy vector evolution via multispecies interbreeding and retargeting of adeno-associated viruses. J. Virol. *82*, 5887–5911.
9. Weinmann, J., Weis, S., Sippel, J., Lenter, M., Lamla, T., and Grimm, D. (2018). Massively Parellel In Vivo Characterization of >150 Adeno-Associated Viral (AAV) Capsids Using DNA/RNA Barcoding and Next-Generation Sequencing. ASGCT 21st Annual Meeting Abstracts, 319–320.
10. Dalkara, D., Byrne, L.C., Klimczak, R.R., Visel, M., Yin, L., Merigan, W.H., Flannery, J.G., and Schaffer, D.V. (2013). In vivo-directed evolution of a new adeno-associated virus for therapeutic outer retinal gene delivery from the vitreous. Sci. Transl. Med. *5*, 189ra76.
11. Adachi, K., Enoki, T., Kawano, Y., Veraz, M., and Nakai, H. (2014). Drawing a high-resolution functional map of adeno-associated virus capsid by massively parallel sequencing. Nat. Commun. *5*, 3075.
12. Koerber, J.T., Maheshri, N., Kaspar, B.K., and Schaffer, D.V. (2006). Construction of diverse adeno-associated viral libraries for directed evolution of enhanced gene delivery vehicles. Nat. Protoc. *1*, 701–706.
13. Deverman, B.E., Pravdo, P.L., Simpson, B.P., Kumar, S.R., Chan, K.Y., Banerjee, A., Wu, W.L., Yang, B., Huber, N., Pasca, S.P., and Gradinaru, V. (2016). Cre-dependent selection yields AAV variants for widespread gene transfer to the adult brain. Nat. Biotechnol. *34*, 204–209.
14. Nonnenmacher, M., van Bakel, H., Hajjar, R.J., and Weber, T. (2015). High capsid-genome correlation facilitates creation of AAV libraries for directed evolution. Mol. Ther. *23*, 675–682.
15. Gao, G., Vandenberghe, L.H., Alvira, M.R., Lu, Y., Calcedo, R., Zhou, X., and Wilson, J.M. (2004). Clades of Adeno-associated viruses are widely disseminated in human tissues. J. Virol. *78*, 6381–6388.
16. Zinn, E., Pacouret, S., Khaychuk, V., Turunen, H.T., Carvalho, L.S., Andres-Mateos, E., Shah, S., Shelke, R., Maurer, A.C., Plovie, E., et al. (2015). In Silico Reconstruction of the Viral Evolutionary Lineage Yields a Potent Gene Therapy Vector. Cell Rep. *12*, 1056–1068.
17. Rabinowitz, J.E., Bowles, D.E., Faust, S.M., Ledford, J.G., Cunningham, S.E., and Samulski, R.J. (2004). Cross-dressing the virion: the transcapsidation of adeno-associated virus serotypes functionally defines subgroups. J. Virol. *78*, 4421–4432.
18. Gurda, B.L., Raupp, C., Popa-Wagner, R., Naumer, M., Olson, N.H., Ng, R., McKenna, R., Baker, T.S., Kleinschmidt, J.A., and Agbandje-McKenna, M. (2012). Mapping a neutralizing epitope onto the capsid of adeno-associated virus serotype 8. J. Virol. *86*, 7739–7751.
19. Samulski, R.J., Chang, L.S., and Shenk, T. (1987). A recombinant plasmid from which an infectious adeno-associated virus genome can be excised in vitro and its use to study viral replication. J. Virol. *61*, 3096–3101.
20. Pacouret, S., Bouzelha, M., Shelke, R., Andres-Mateos, E., Xiao, R., Maurer, A., Mevel, M., Turunen, H., Barungi, T., Penaud-Budloo, M., et al. (2017). AAV-ID: A Rapid and Robust Assay for Batch-to-Batch Consistency Evaluation of AAV Preparations. Mol. Ther. *25*, 1375–1386.
21. D'Costa, S., Blouin, V., Broucque, F., Penaud-Budloo, M., François, A., Perez, I.C., Le Bec, C., Moullier, P., Snyder, R.O., and Ayuso, E. (2016). Practical utilization of recombinant AAV vector reference standards: focus on vector genomes titration by free ITR qPCR. Mol. Ther. Methods Clin. Dev. *5*, 16019.
22. Ayuso, E., Blouin, V., Lock, M., McGorray, S., Leon, X., Alvira, M.R., Auricchio, A., Bucher, S., Chtarto, A., Clark, K.R., et al. (2014). Manufacturing and characterization of a recombinant adeno-associated virus type 8 reference standard material. Hum. Gene Ther. *25*, 977–987.
23. Livak, K.J., and Schmittgen, T.D. (2001). Analysis of relative gene expression data using real-time quantitative PCR and the 2(-Delta Delta C(T)) Method. Methods *25*, 402–408.
24. Chang, L.S., Shi, Y., and Shenk, T. (1989). Adeno-associated virus P5 promoter contains an adenovirus E1A-inducible element and a binding site for the major late transcription factor. J. Virol. *63*, 3479–3488.
25. Lisowski, L., Dane, A.P., Chu, K., Zhang, Y., Cunningham, S.C., Wilson, E.M., Nygaard, S., Grompe, M., Alexander, I.E., and Kay, M.A. (2014). Selection and evaluation of clinically relevant AAV variants in a xenograft liver model. Nature *506*, 382–386.

26. Earley, L.F., Powers, J.M., Adachi, K., Baumgart, J.T., Meyer, N.L., Xie, Q., Chapman, M.S., and Nakai, H. (2017). Adeno-associated Virus (AAV) Assembly-Activating Protein Is Not an Essential Requirement for Capsid Assembly of AAV Serotypes 4, 5, and 11. J. Virol. *91*, e01980-16.

27. Grosse, S., Penaud-Budloo, M., Herrmann, A.K., Börner, K., Fakhiri, J., Laketa, V., Krämer, C., Wiedtke, E., Gunkel, M., Ménard, L., et al. (2017). Relevance of Assembly-Activating Protein for Adeno-associated Virus Vector Production and Capsid Protein Stability in Mammalian and Insect Cells. J. Virol. *91*, e01198-17.

28. Dudek, A.M., Pillay, S., Puschnik, A.S., Nagamine, C.M., Cheng, F., Qiu, J., Carette, J.E., and Vandenberghe, L.H. (2018). An Alternate Route for Adeno-associated Virus (AAV) Entry Independent of AAV Receptor. J. Virol. *92*, e02213-17.

29. Maurer, A.C., Pacouret, S., Cepeda Diaz, A.K., Blake, J., Andres-Mateos, E., and Vandenberghe, L.H. (2018). The Assembly-Activating Protein Promotes Stability and Interactions between AAV's Viral Proteins to Nucleate Capsid Assembly. Cell Rep. *23*, 1817–1830.

30. Gao, G.P., Alvira, M.R., Wang, L., Calcedo, R., Johnston, J., and Wilson, J.M. (2002). Novel adeno-associated viruses from rhesus monkeys as vectors for human gene therapy. Proc. Natl. Acad. Sci. USA *99*, 11854–11859.

OMTM, Volume *17*

Supplemental Information

Cross-Packaging and Capsid Mosaic

Formation in Multiplexed AAV Libraries

Pauline F. Schmit, Simon Pacouret, Eric Zinn, Elizabeth Telford, Fotini Nicolaou, Frédéric Broucque, Eva Andres-Mateos, Ru Xiao, Magalie Penaud-Budloo, Mohammed Bouzelha, Nicolas Jaulin, Oumeya Adjali, Eduard Ayuso, and Luk H. Vandenberghe

Supplemental Material

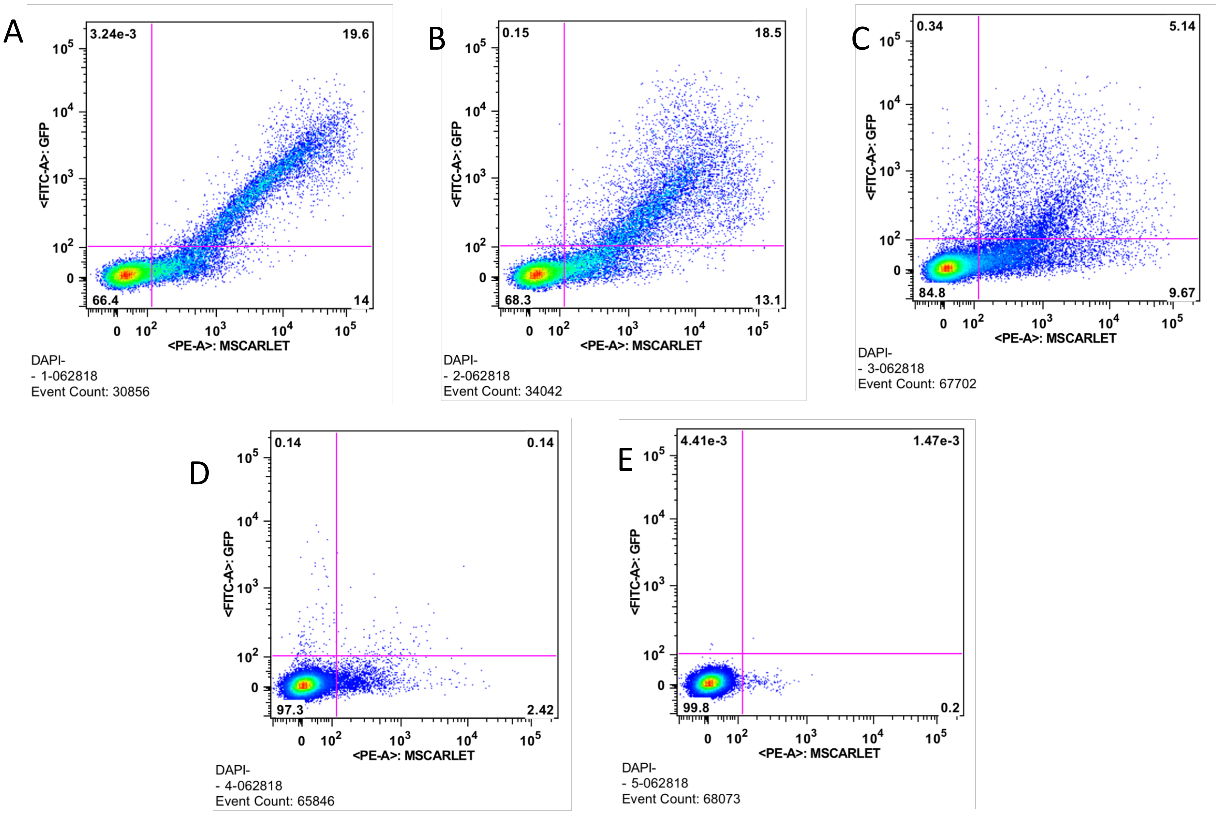

Figure S1: Flow cytometry of dually-transfected cell populations. A. HEK293 cells co-transfected with 1.3 ug each of ITR-flanked CMV.eGFP plasmid, CMV. mScarlet plasmid, and ITR-free AAV2/8 Rep/Cap production plasmids, in addition to 2.6 ug deltaF6 and a promoter-free dummy plasmid to normalize total DNA transfected. Cells were harvested after 48 hours and subjected to flow cytometry. Plots shown consist of single live cells. **B.** Cells transfected and analyzed as in **A**, but with 390 ng total AAV8/eGFP/mScarlet mixture. **C.** Cells transfected and analyzed as in **A**, but with 39 ng total AAV8/eGFP/mScarlet mixture. **D.** Cells transfected and analyzed as in **A**, but with 3.9 ng total AAV8/eGFP/mScarlet mixture. **E.** Cells transfected and analyzed as in **A**, but with 390 pg total AAV8/eGFP/mScarlet mixture.

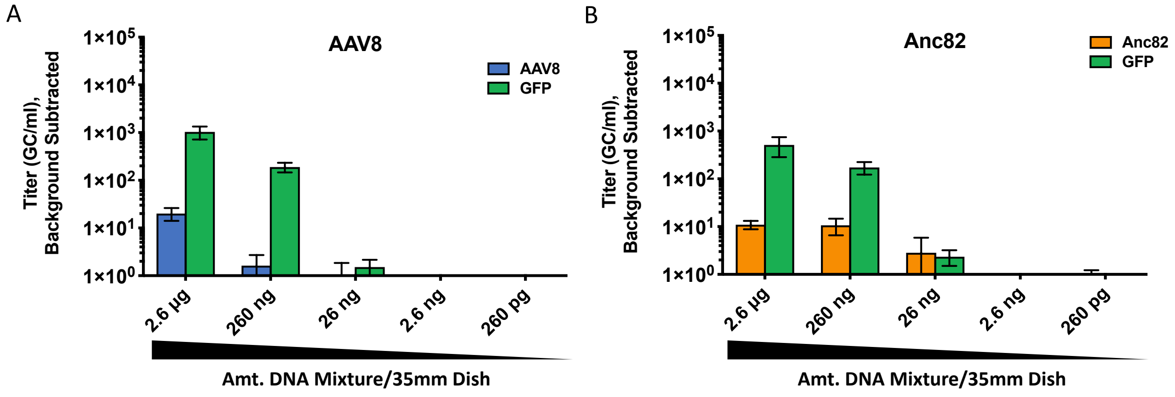

Figure S2: Effects of dilution on negative control 'libraries'. A. qPCR titration of AAV8/GFP 'libraries' created through transfection of dilutions of recombinant pAAVector.2/8 backbone along with GFP/luciferase. Titers of non-producing negative control preps were used to normalize values. Values represent average of three independent experiments, and error bars represent SEM. **B**. Same methods and computations as **A**, only using pAAVector.2/Anc82 backbone.

A

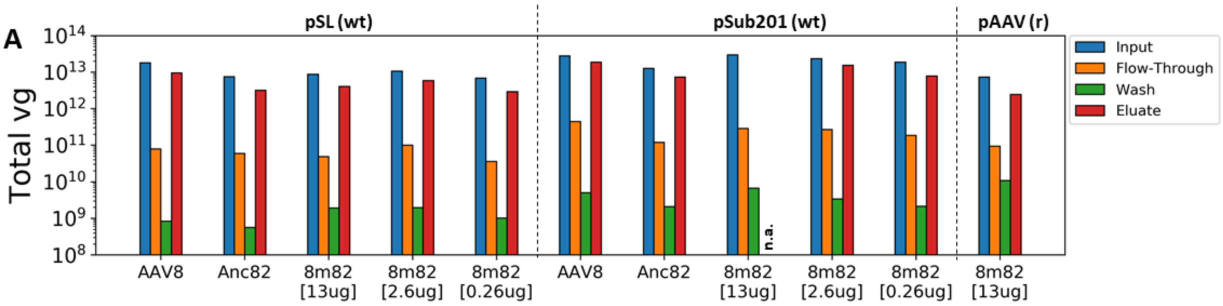

B

Yields (%)	AAV8	Anc82	AAV8m82 [13ug]	AAV8m82 [2.6ug]	AAV8m82 [0.26ug]
pSL-AAV	52.6	42.2	46.7	55.4	42.6
pSub201-AAV	68.2	58.5	n.a.	66.5	42.5
pAAV-AAV	-	-	33.2	-	-

Fig S3: Assessment of the bias of our library purification method. A. vg levels, quantified in the input, flow-through, wash and elution fraction by ITR2-free qPCR. **B**. vg recovery in the elution fractions.

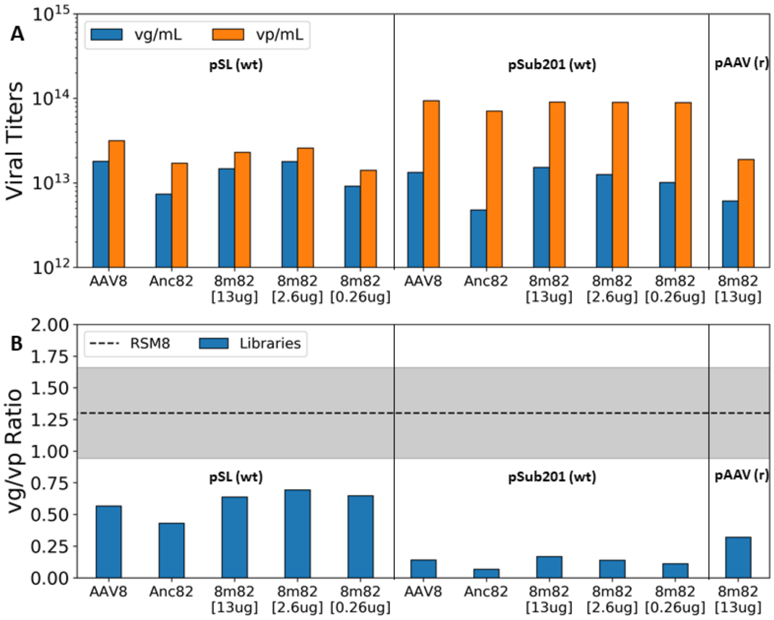

C

Preparation	%Full Capsids
RSM8	~100
pSL-AAV8	43.68
pSL-Anc82	33.24
pSL-AAV8m82 [13ug]	49.16
pSL-AAV8m82 [2.6ug]	53.37
pSL-AAV8m82 [0.26ug]	49.97
pSub201-AAV8	10.87
pSub201-Anc82	5.20
pSub201-AAV8m82 [13ug]	13.02
pSub201-AAV8m82 [2.6ug]	10.78
pSub201-AAV8m82 [0.26ug]	8.68
pAAV-AAV8m82 [13ug]	24.66

Fig S4: Quantification of vg levels, vp levels, and full/empty particle ratios in concentrated, buffer-exchanged AAV library preparations. A. vg and vp titers, measured by ITR2-free qPCR and SDS- PAGE densitometry, respectively. **B**. vg/vp ratios calculated for every library preparation. The mean experimental ratio obtained for the RSM8 (~100% full particles) is represented in dashed line. The shaded gray area represents the standard deviation from the mean (n=3 independent qPCR and ELISA assays). **C**. Assessment of the fraction of full particles in AAV library preparations.

Well	Preparation
1	pSL-AAV8
2	pSL-Anc82
3	pSL-AAV8m82 [13ug]
4	pSL-AAV8m82 [2.6ug]
5	pSL-AAV8m82 [0.26ug]
6	pSub201-AAV8
7	pSub201-Anc82
8	pSub201-AAV8m82 [13ug]
9	pSub201-AAV8m82 [2.6ug]
10	pSub201-AAV8m82 [0.26ug]
11	pAAV-AAV8m82 [13ug]

A

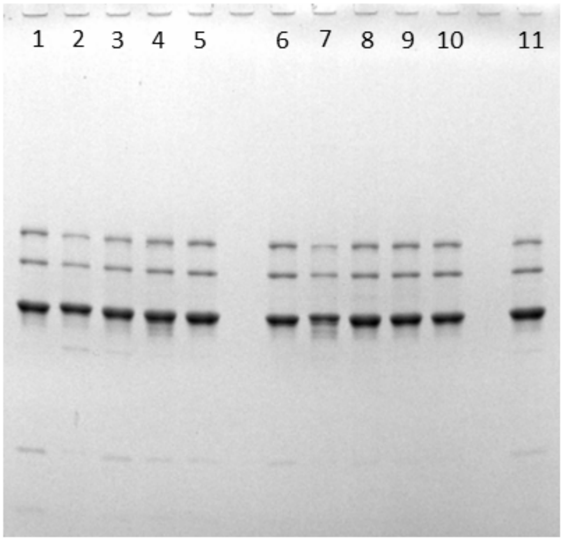

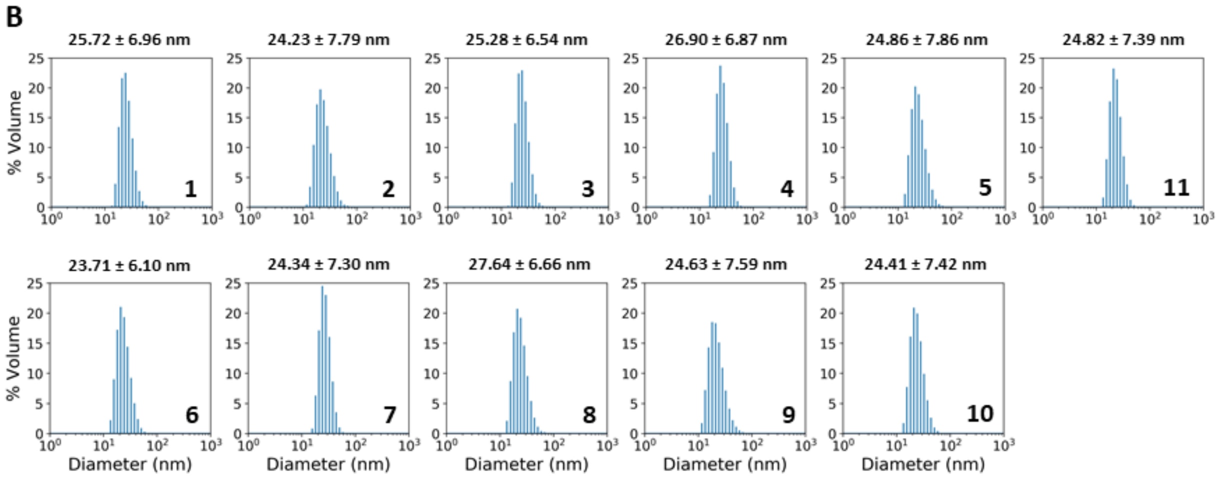

Fig S5: Evaluation of AAV library purity and size homogeneity. **A**. SDS-PAGE analysis of AAV library purity, at $1.4x10^{11}$ vp/well (Coomassie blue staining). **B**. Dynamic Light Scattering (DLS) analysis of AAV library size homogeneity.

.

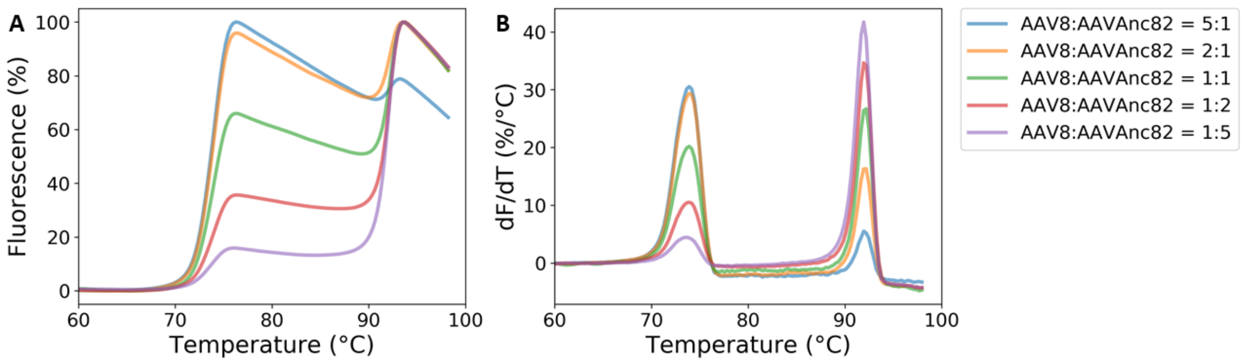

Fig S6. Impact of AAV8 and Anc82 relative vp levels on DSF. Normalized fluorescence (**A**) and fluorescence derivative (**B**) obtained at various AAV8:Anc82 stoichiometries. Preparations were all analyzed at 7.7x10^{11} vp/well.

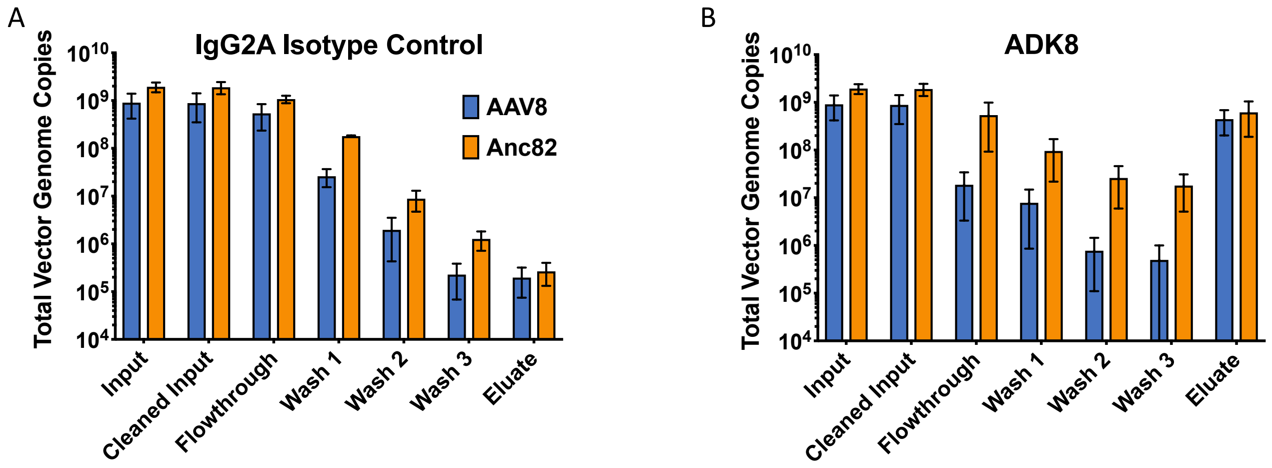

Figure S7: Immunoprecipitation of AAV8 and Anc82 particles. A. Pulldowns of AAV8 or Anc82 particles with a mouse IgG2A Isotype control antibody. Crude preps were applied to antibody coated Protein A/G agarose beads. Fractions were taken at each stage and titered using AAV8 or Anc82 capsid specific Taqman probes. Error bars represent SEM of three independent experiments. **B.** Pulldowns of AAV8 or Anc82 particles with a ADK8. Methods and computations are identical to **A.**

Figure S8: Derivation of mathematical model describing packaging and mosaicism

For each vector population analyzed, there is cDNA for 2 samples (ADK8 treated and isotype treated). Each of these cDNA samples is measured by RT-qPCR for AAV8 capsid, Anc82 capsid, and beta-actin. To calculate r_{total} for a given genome species, 4 values are required:

Isotype Treated cDNA	ADK8 Treated cDNA
$Ct_{capsid, isotype}$	$Ct_{capsid,ADK8}$
$Ct_{actin, isotype}$	$Ct_{actin,ADK8}$

Calculate dCt values to normalize for cDNA quality:

$$\Delta Ct_{isotype} = Ct_{capsid,isotype} - Ct_{actin,isotype} \tag{1}$$

$$\Delta Ct_{ADK8} = Ct_{capsid,ADK8} - Ct_{actin,ADK8} \tag{2}$$

Calculate ddCt value:

$$\Delta\Delta Ct = \Delta Ct_{ADK8} - \Delta Ct_{isotype} \tag{3}$$

Convert to fold change:

$$\text{Fold Change} = 2^{-\Delta\Delta Ct} = r_{total} \tag{4}$$

Where r_{total} is the fold change in cDNA abundance with ADK8 treatment, otherwise known as the **neutralization coefficient** of the vector population

$$r_{total} = \frac{\text{cDNA following ADK8 treatment}}{\text{cDNA following isotype control treatment}} = \frac{Y_{total}}{X_{total}} \tag{5}$$

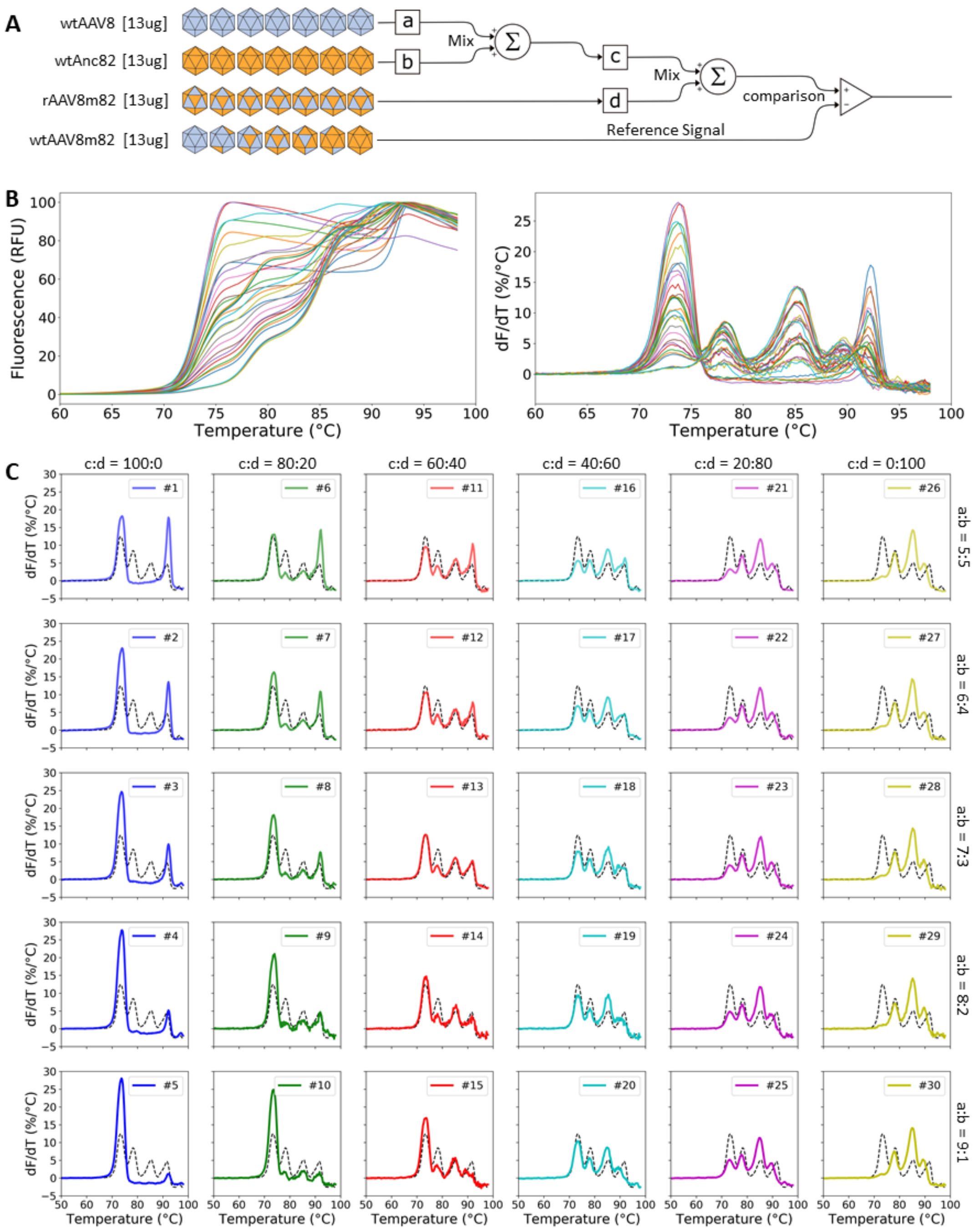

Fig S9. Recapitulation of pSub201-AAV8m82 [13ug] fluorescence signal through analysis of a mix of pSub201-AAV8, pSub201-Anc82 and rAAV8m82 [13ug] particles. **A**. Graphical representation of the different wtAAV8:wtAnc82:rAAV8m82 stoichiometries tested to recapitulate wtAAV8m82 fluorescence signal. The ratio a:b varied between 5:5 and 9:1, based on our previous observation that AAV8 produced better than Anc82. The ratio c:d varied between 100:0 and 0:100. (**B**, **C**). Fluorescence and derivative fluorescence signals obtained for the entire set of preparations, analyzed at $3x10^{11}$ vp/well. **D**. Individual derivative fluorescence signals, overlapped with the reference signal, obtained for pSub201-AAV8m82[13ug] (dashed line).

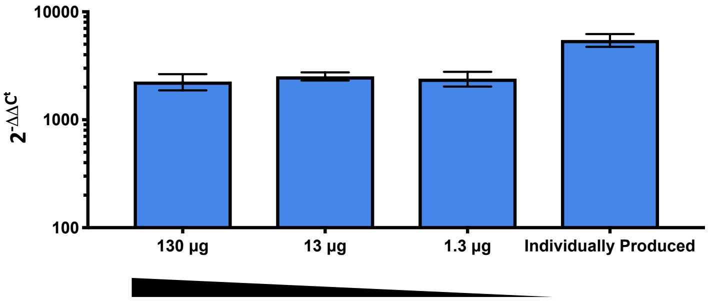

Figure S10: Relative abundance of transgene DNA in liver gDNA of mice injected with barcoded libraries. Liver gDNA of injected mice was probed for Tfrc and CMV DNA sequences to measure relative abundance of transgene DNA in livers.

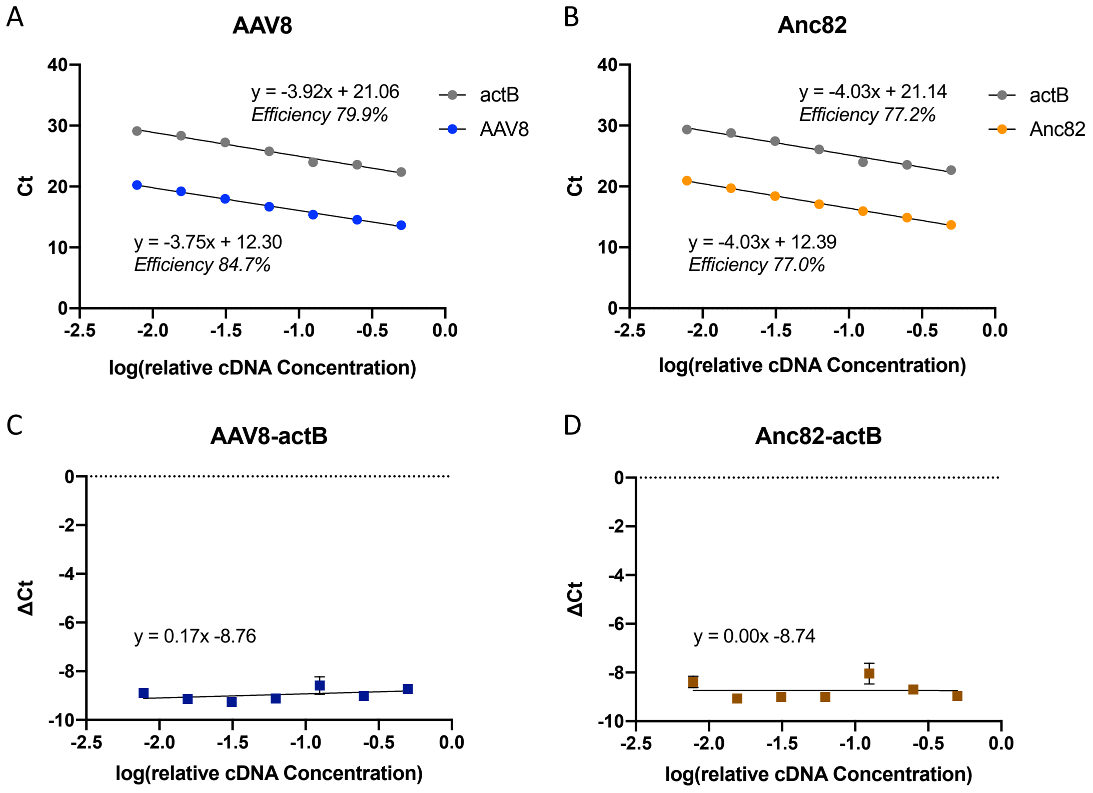

Figure S11: Efficiency of Taqman probes used in RT-qPCR. A. Relative quantification of beta actin and AAV8 capsid in serial dilutions of cDNA from HEK293 cells transfected with pAAVector2/8 using PEI. Assay efficiency is determined by the slope of the line. **B.** Similar to **A**, but using cDNA from cells transfected with pAAVector2/Anc82. **C.** Delta Ct calculations using data from **A** across cDNA concentrations. **D.** Delta Ct calculations using data from **B** across cDNA concentrations.

Table S1. **Summary of the melting temperatures** obtained for the SL (top) and pSub201 (bottom) AAV library preparations. (n=3 independent runs).

A

pSL-AAV	AAV8+Anc82	AAV8m82 [13ug]	AAV8m82 [2.6ug]	AAV8m82 [0.26ug]
Tm1 (°C)	73.37 ± 0.15	73.2 ± 0.0	73.46 ± 0.0	73.64 ± 0.15
Tm2 (°C)	-	77.98 ± 0.15	78.48 ± 0.58	77.98 ± 0.15
Tm3 (°C)	-	85.19 ± 0.0	85.36 ± 0.30	84.93 ± 0.26
Tm4 (°C)	91.79 ± 0.15	91.19 ± 0.0	91.79 ± 0.15	91.96 ± 0.0

B

pSub201-AAV	AAV8+Anc82	AAV8m82 [13ug]	AAV8m82 [2.6ug]	AAV8m82 [0.26ug]
Tm1 (°C)	73.46 ± 0.0	73.11 ± 0.15	73.37 ± 0.15	73.46 ± 0.0
Tm2 (°C)	-	77.89 ± 0.0	77.98 ± 0.15	78.07 ± 0.31
Tm3 (°C)	-	84.84 ± 0.15	85.10 ± 0.54	84.50 ± 0.65
Tm4 (°C)	91.61 ± 0.15	91.18 ± 0.45	91.44 ± 0.0	91.61 ± 0.15

Table S2: Oligonucleotides Used for Cloning

Primer	Orientation	Sequence
**KpnI-r2cAnc82**	Forward	5'-cagacaggtaccaaaacaaatgttctcg-3'
**r2cAnc82-AgeI**	Reverse	5'-gaattaaccggtttattgattaacaagcaaactagtttacagattacgg-3'
**HindIII-r2c8-t2a-mCherry**	Forward	5'-gacgcggaagcttcgatcaactac-3'
**r2c8-t2a-mCherry-SpeI**	Reverse	5'-taagcaactagtctacttgtacagctcgtccatgccgc-3'

Table S3: Transfection Mixes for Production of AAV8/Anc82 Capsid Libraries for DSF

System	Transfection Mix (for one 15-cm dish; X = 13ug, 2.6 ug, 1.3ug, 260 ng or 130 ng)
**pSub201**	26 ug ΔF6 Helper + X/2 ug pSub201-r2c8 + X/2 ug pSub201-r2cAnc82 + (26-X) ug pSEAP2
**pSL**	26 ug ΔF6 Helper + X/2 ug pSL-r2c8 + X/2 ug pSL-r2cAnc82 + 13 ug pRep2 + (13-X) ug pSEAP2
**pAAV**	26 ug ΔF6 Helper + 6.5 ug pAAV-r2c8 + 6.5 ug pAAV-r2cAnc82 + 13 ug pTransgene

Table S4: Transfection Mixes for Production of AAV8/Anc82 Capsid Libraries for Cross-packaging Analysis. For the sake of consistency with DSF libraries, conditions are labeled by the amount of total pAAV plasmid that would be transfected in a recombinant AAV production. Since the sizes of pSL and pSub201 constructs vary, the actual quantities of each plasmid in the mixture were adjusted to ensure equimolar *cap* amounts across conditions, and pDummy plasmid was added to normalize the total ug quantity of DNA transfected

System	ΔF6 Helper	AAV8 Plasmid	Anc82 Plasmid	pRep	pDummy (promoter free plasmid)
**pSL – 13 ug**	26 ug	5.7 ug	5.7 ug	9.91 ug	
**pSL – 1.3 ug**	26 ug	570 ng	570 ng	9.91 ug	10.3 ug
**pSL – 130 ng**	26 ug	57 ng	57 ng	9.91 ug	11.3 ug
**pSub201 – 13 ug**	26 ug	7.4 ug	7.4 ug		6.6 ug
**pSub201 – 1.3 ug**	26 ug	740 ng	740 ng		19.8 ug
**pSub201 – 130 ng**	26 ug	74 ng	74 ng		21.2 ug

Table S5: Oligonucleotides Used for qPCR

	Forward	Reverse	Probe
CMV2	5'-CATCTACGTATTAGTCATCGCTATTACCA-3'	5'-GAAATCCCCGTGAGTCAAACC-3'	5'-TCAATGGGCGTGGATAG-3'
eGFP	5'-AGCAAAGACCCCAACGAGAA-3'	5'-GGCGGCGGTCACGAA-3'	5'-CGCGATCACATGGTCCTGCTGG-3'
AAV8 Capsid	5'-TTTGCCTGGACTGCTGGG-3'	5'-TGCCAAAAATCAGGATCCCGTTAC-3'	5'-AAGAAATTCATTGGCTAATCCTGGCATCG-3'
Anc82 Capsid	5'-CTACGGGAGGCACAGCGG-3'	5'-GTCCAGGCAGCCAGTTTTTG-3'	5'-CCAGACGTTGCAGTTTTCTCAGGCC-3'
Hs Actin	5'-ACAGAGCCTCGCCTTTG-3'	5'-CCTTGCACATGCCGGAG-3'	5'-TCATCCATGGTGAGCTGGCGG-3'
Library BC Amplification	5'-TCGTCGGCAGCGTCAGATGTGTATAAGAGACAGGAGGTCTATATAAGCAGAGCTGGTTTAGTGAACCGT-3'	5'-GTCTCGTGGGCTCGGAGATGTGTATAAGAGACAGGACGAGAACATTTGTTTTGGTACCTGTCTGCGTAG-3'	

Supplemental Methods:

FACS: Individual wells of 6 well plates containing HEK293 cells at approximately 90% confluency were transfected with a total of 6.5 μg DNA using PEI Max, including 2.6 μg deltaF6, between 3.9 μg-390 pg of an equimolar mixture of pAAVector2/8, ITR-CMV.eGFP plasmid, and ITR-CMV.mScarlet, and a dummy plasmid to control for total DNA added. Cells were harvested after 48 hours by trypsinization, washed with FACS buffer (1X DPBS (Corning) supplemented with 5% fetal bovine serum and 2% EDTA), and resuspended in FACS buffer before being subjected to flow cytometry.

SDS-PAGE Densitometry: For quantification of vp levels, SDS-PAGE were run as described in methods. STR8, analyzed three times by ELISA using the RSM8, was used to generate a standard curve. STR8 was first subjected to 1.5-fold serial dilution, between $3.32x10^{13}$ pt/mL (1:1) and $4.37x10^{12}$ pt/mL (1:7.59375). 10 μL of each standard were mixed with 5 μL PBD2+ and 5 μL 1X NuPAGE lithium dodecyl sulfate (LDS) sample buffer (NP007, ThermoFisher) (Boston) or 1X Laemmli sample buffer (161-0747, Biorad) (Nantes). Diluted unknown samples (dilution factors determined empirically) were subjected to the same treatment. 20 μL SDS-PAGE mixes were incubated for 10 min at 99 °C and subjected to a quick spin. 15 μL of each mix were further loaded on a 10-well polyacrylamide gel (see above for the references of the gels used in Nantes and Boston), and run for 2.5-3 h at 100 V. Gels were stained as described above and imaged. For the standard and unknown samples, the pixel density of VP3 was measured using Fiji. The standard curve was further fitted using a second order polynomial function, used to calculate the vp levels in each unknown sample based on their VP3 pixel density value.

Immunoprecipitations: Crude preps of vector produced from pSL-AAV8 or pSL-Anc82 were diluted to 7.50E+09 GC/ml and treated with either mouse IgG2A kappa or ADK8 antibody at a final dilution of 1:1250. Immunoprecipitations were preformed using Pierce™ Protein A/G Agarose (20423, Thermo Fisher). Vector Particles were eluted in 0.2 M glycine, pH 2.8 and neutralized in equal volumes of 100 mM Tris pH 8.5. All fractions were collected, and DNAse I-protected total vector genomes were quantified using TaqMan probes against AAV8 or Anc82 and the Applied Biosystems 7500 Real-time PCR System.

Liver Transgene Abundance Assay: Liver gDNA isolated from injected mice was quantified using a TaqMan™ Tfrc Copy Number Reference Assay (4458366, Thermo Fisher) and a TaqMan assay recognizing CMV2 promoter DNA sequence. 25 ng total gDNA was measured per 25 μl reaction. qPCR was carried out using the Applied Biosystems 7500 Real-time PCR System.

TaqMan Probe Efficiency Assay: Individual wells of 6 well plates containing HEK293 cells at approximately 90% confluency were transfected with 2.6 μg deltaF6 and with either 1.3 μg pAAVector2/8 or 1.3 μg pAAVector2/82 using PEI Max. Cells were harvested in TriZol and RNA was extracted and cDNA synthesized as described in methods. Serial dilutions of cDNA were used as input for RT-qPCR reactions.
